# Evolution of the facial musculature in basal ray-finned fishes

**DOI:** 10.1186/s12983-018-0285-6

**Published:** 2018-10-25

**Authors:** Aléssio Datovo, Pedro P Rizzato

**Affiliations:** 1 Laboratório de Ictiologia, Museu de Zoologia da Universidade de São Paulo (MZUSP), Av. Nazaré, 481, São Paulo, 04263-000 SP Brazil; 2 Laboratório de Ictiologia de Ribeirão Preto (LIRP), FFCLRP, Universidade de São Paulo, Av. Bandeirantes, 3900, Ribeirão Preto, SP 14040-901 Brazil

**Keywords:** Morphology, Myology, Musculature, Cheek muscles, Adductor mandibulae, Constrictor dorsalis, Osteichthyes, Actinopterygii, Teleostei, Neopterygii

## Abstract

**Background:**

The facial musculature is a remarkable anatomical complex involved in vital activities of fishes, such as food capture and gill ventilation. The evolution of the facial muscles is largely unknown in most major fish lineages, such as the Actinopterygii. This megadiverse group includes all ray-finned fishes and comprises approximately half of the living vertebrate species. The Polypteriformes, Acipenseriformes, Lepisosteiformes, Amiiformes, Elopiformes, and Hiodontiformes occupy basal positions in the actinopterygian phylogeny and a comparative study of their facial musculature is crucial for understanding the cranial evolution of bony fishes (Osteichthyes) as a whole.

**Results:**

The facial musculature of basal actinopterygians is revised, redescribed, and analyzed under an evolutionary perspective. We identified twenty main muscle components ontogenetically and evolutionarily derived from three primordial muscles. Homologies of these components are clarified and serve as basis for the proposition of a standardized and unifying myological terminology for all ray-finned fishes. The evolutionary changes in the facial musculature are optimized on the osteichthyan tree and several new synapomorphies are identified for its largest clades, including the Actinopterygii, Neopterygii, and Teleostei. Myological data alone ambiguously support the monophyly of the Holostei. A newly identified specialization constitutes the first unequivocal morphological synapomorphy for the Elopiformes. The myological survey additionally allowed a reinterpretation of the homologies of ossifications in the upper jaw of acipenseriforms.

**Conclusions:**

The facial musculature proved to be extremely informative for the higher-level phylogeny of bony fishes. These muscles have undergone remarkable changes during the early radiation of ray-finned fishes, with significant implications for the knowledge of the musculoskeletal evolution of both derived actinopterygians and lobe-finned fishes (Sarcopterygii).

## Background

The skeletal musculature exhibits an extraordinary diversification across multiple fish lineages and constitutes a rich source of phylogenetic information [1–5]. Nevertheless, this anatomical system has been traditionally neglected in most evolutionary studies [6]. Most contributions to fish myology focused on the functional aspects of this system in only a few species, with little or no concern about muscle homologies and their phylogenetic implications for higher taxa. The facial musculature is a remarkable myological complex that includes the most superficial and easily accessible muscles of the cheek. These muscles are involved in some of the most vital activities of fishes, such as food capture and gill ventilation. A notable variation in the facial musculature is observed across the major lineages of fishes, but the current knowledge about the evolution of this anatomical complex is extremely deficient.

Ray-finned fishes compose the class Actinopterygii, the largest radiation of aquatic vertebrates with over 32,000 species [7, 8] and one of the greatest challenges of vertebrate systematics [9–11]. Four orders, with only 49 extant species, occupy the basalmost region of the actinopterygian tree: Polypteriformes (bichirs and reedfish; 12 spp.), Acipenseriformes (sturgeons and paddlefishes; 29 spp.), Lepisosteiformes (gars; 7 spp.), and Amiiformes (bowfin; 1 sp.) [7, 10]. These orders form clades that are successive sister groups of the Teleostei, a megadiverse taxon that comprises the vast majority of extant fishes. The Elopiformes and Hiodontiformes include generalized representatives of the two basalmost teleost lineages, the Elopomorpha and Osteoglossomorpha, respectively [10, 12]. Identification of the primitive features in the facial musculature of these basal actinopterygians is crucial for understanding the major events in the evolution of this system across the Osteichthyes. Such an effort should additionally provide the basis for identifying informative myological characters at different levels of the bony-fish tree.

This study presents a detailed comparative survey of the facial musculature of representatives of eight families of basal actinopterygians. A homology-driven terminology for the actinopterygian facial muscles is proposed and a comprehensive synonymy is provided for the myriad of muscle names employed in the most important previous studies. The phylogenetic implications of the major changes in the facial musculature are analyzed and several new synapomorphies for major actinopterygian clades are advanced.

## Results

We present in this section detailed anatomical descriptions of the facial muscles of the examined species (Table 1), followed by a list of synonyms for each muscle component reported in the most important previous studies. This facilitates access to information in prior publications within the context of our terminology. Justifications for the inferences of homologies and the consequent muscle terminology herein applied are discussed in the section “Homologies of the facial muscles in the Actinopterygii”.

**Table 1:** Material examined. Abbreviations: c&s, cleared and stained; mus, muscle dissection; UD, unavailable data. Additional comparative material of Teleostei listed in Datovo & Vari [6, 28] and Datovo et al. [83]

Order	Family	Species	Catalog number	Examined specimens	Preparation	Standard length (mm)
Acipenseriformes	Acipenseridae	*Acipenser fulvescens*	MZUSP 48364	1	mus	121
			VIMS 17717	1	c&s	141
			VIMS 35591	1	mus	206
			VIMS 35592	1	c&s	87
			VIMS 35593	3	c&s	85–111
	Polyodontidae	*Polyodon spathula*	VIMS 19827	3	c&s	219–263
			VIMS 35594	1	mus	245
Amiiformes	Amiidae	*Amia calva*	USNM 64338	1	mus	176
			VIMS 35717	1	mus	UD (head only)
Elopiformes	Elopidae	*Elops lacerta*	MZUSP 84787	1	mus	111
	Megalopidae	*Megalops cyprinoides*	USNM 102685	1	mus	106
Hiodontiformes	Hiodontidae	*Hiodon alosoides*	VIMS 35598	1	mus	104
		*Hiodon tergisus*	USNM 167970	1	mus	81
Lepisosteiformes	Lepisosteidae	*Lepisosteus osseus*	LIRP 13576	1	mus	171
			VIMS 13559	2	c&s	73–100
		*Lepisosteus platostomus*	USNM 54983	1	mus	201
Polypteriformes	Polypteridae	*Calamoichthys calabaricus*	LIRP 13572	1	c&s	233
			LIRP 4572	1	mus	235
			USNM 380263	2	mus	257–267
		*Polypterus senegalus*	LIRP 13548	1	c&s	87
			LIRP 14021	1	c&s	122
			USNM 224817	1	mus	232
			USNM 229760	1	c&s	137
				1	mus	108
			USNM 395402	1	mus	214

### Polypteriformes

#### Polypterus senegalus (Figs. 1 and 2)

The adductor mandibulae has three well-differentiated muscle segments. The most superficial one is the segmentum facialis, located ventrolateral to the constrictor mandibularis dorsalis. The segmentum facialis has a pars ricto-malaris and a pars stegalis partially separated from each other at both their origins and insertions. The ricto-malaris originates from the preopercle, hyomandibula, metapterygoid, posterior borders of the quadrate and palatoquadrate cartilage, and a small area (just anterior to the hyomandibula) of the hyopalatine membrane. In some specimens, a partial separation between rictalis and malaris is observed at their medial surfaces. The ricto-malaris inserts on the angular and coronoid process of the prearticular. Origin of the stegalis is from the quadrate and posterior portion of the palatoquadrate cartilage. The posteriormost fibers of the stegalis insert directly on the angular, but most fibers of this section converge to a meckelian tendon that anchors to the angular. Anteriorly, the meckelian tendon is continuous with the mandibular tendon, which collects the fibers of the segmentum buccalis (see below).

**Fig. 1:**
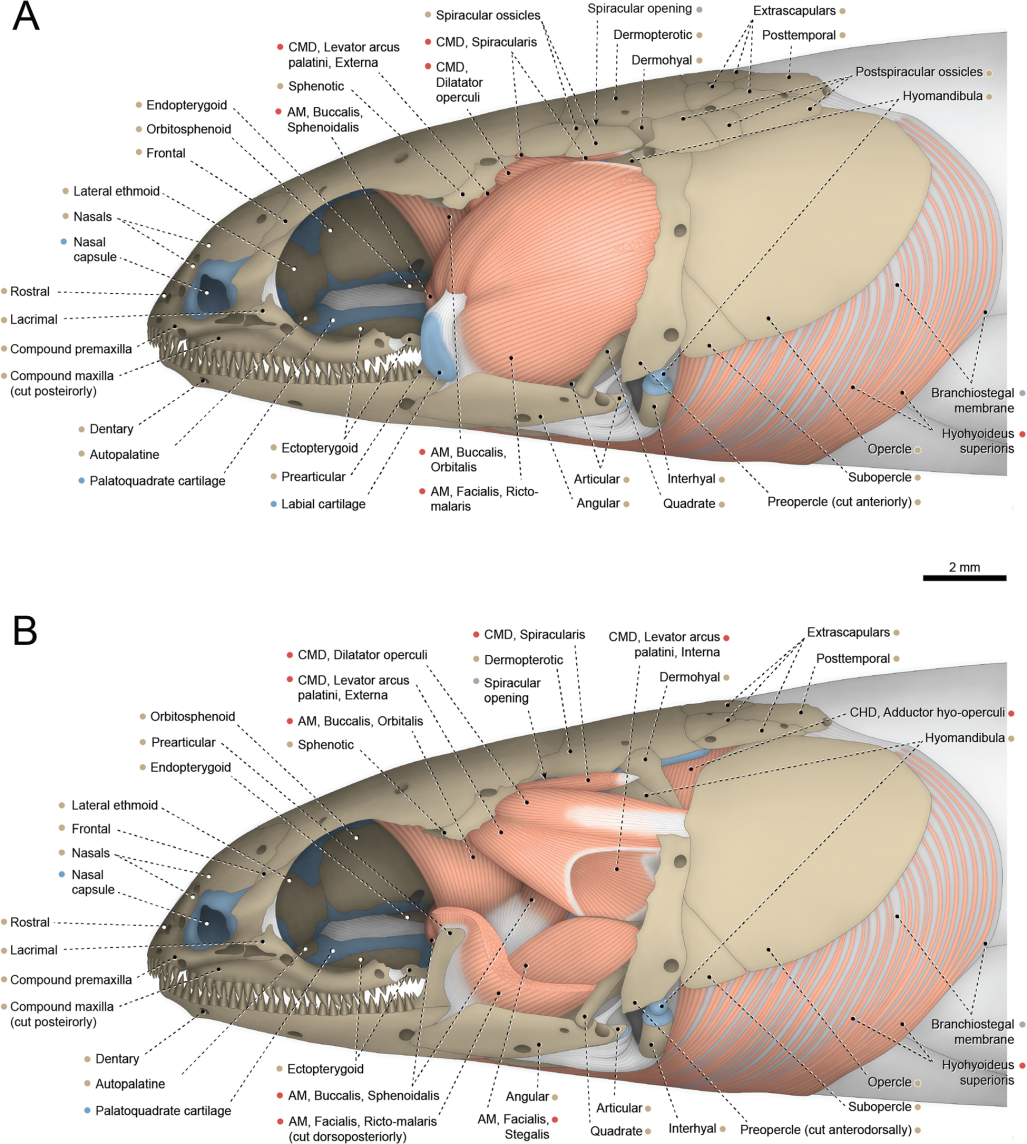
Cranial musculoskeletal system of *Polypterus senegalus*, USNM 229760; left lateral view; first nasal (= os terminale), prespiracular ossicles, gular plate, postorbital (= postinfraorbital), posterior portion of compound maxilla, and cheek plate of preopercle removed. **a** Superficial musculature. **b** Deeper musculature; spiracular and postspiracular ossicles, labial cartilage, and dorsal portions of preopercle and pars ricto-malaris of adductor mandibulae additionally removed. Abbreviations: AM, adductor mandibulae; CHD, constrictor hyoideus dorsalis; CMD, constrictor mandibularis dorsalis. Color code: Salmon, muscle; sand, bone; blue, cartilage; white, tendon or ligament (regular connective tissue); dark grey, irregular connective tissue

**Fig. 2:**
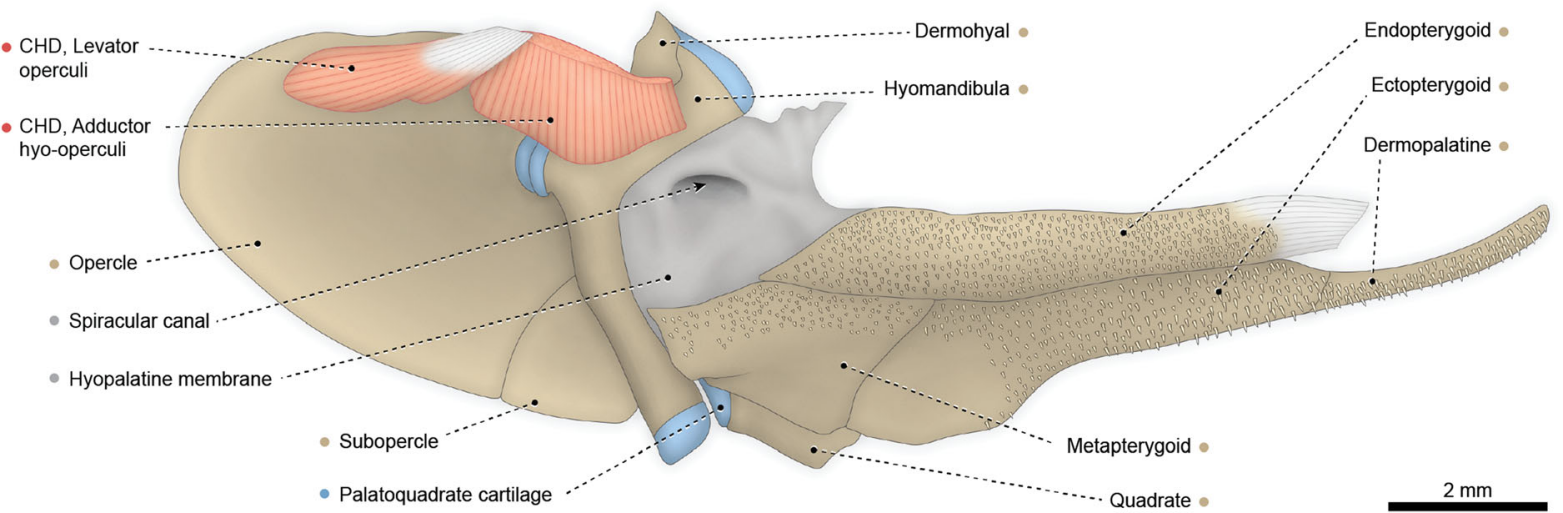
Hyopalatine arch, opercular series, and associated constrictor hyoideus dorsalis of *Polypterus senegalus*, USNM 229760; left medial view. Abbreviation: CHD, constrictor hyoideus dorsalis. Color code: Salmon, muscle; sand, bone; blue, cartilage; white, tendon or ligament (regular connective tissue); dark grey, irregular connective tissue

Medial to the segmentum facialis lies the segmentum buccalis of the adductor mandibulae. This muscle segment is dorsally subdivided into a pars sphenoidalis and a pars orbitalis. The former originates completely medial to the levator arcus palatini, from the orbitosphenoid and parasphenoid. Origin of the pars orbitalis is from the frontal, sphenotic, and posterior portion of the supraorbital cartilage (associated with the frontal). Towards insertion, the orbitalis and sphenoidalis merge to each other and converge to a strong mandibular tendon that inserts on the angular.

The segmentum mandibularis (not illustrated) is very small and concealed laterally by the angular and medially by the prearticular. Origin is from the anteromedial portion of the mandibular tendon and insertion is on the angular, prearticular, and Meckel’s cartilage.

The constrictor mandibularis dorsalis is subdivided into distinct muscles. The dorsalmost is the spiracularis, a small muscle originating from the contact area between the sphenotic, frontal, and dermopterotic. The muscle runs posteriorly and inserts medially on the lateral wall of the spiracular tube and posterolaterally on the internal face of the spiracular ossicles. The remainder of the constrictor mandibularis dorsalis is completely separated from the spiracularis and originates from the ventral region of the sphenotic. A partial separation between dilatator operculi and levator arcus palatini is observed along their lateral faces. Posteriorly, the fibers of the dilatator operculi separate from the surrounding muscle fibers and pass through a fenestra between the dorsal portions of the preopercle and hyomandibula. Insertion of the dilatator operculi is on the anterodorsal region of the opercle. Towards insertion, the levator arcus palatini partially subdivides into a smaller pars externa and a larger pars interna. Fibers of the former converge to an arched insertional tendon that anchors anteriorly to the metapterygoid and posteriorly to the hyomandibula. Insertion of the pars interna of the levator arcus palatini is on the endopterygoid, metapterygoid, palatoquadrate cartilage, hyomandibula and, most extensively, on the hyopalatine membrane forming the lateral wall of the spiracular canal.

The constrictor hyoideus dorsalis is completely separated into two subdivisions, both arising from the lateral region of the opisthotic (= epioccipital). The anterior division is the adductor hyo-operculi, which corresponds to the complex adductor hyomandibulae plus adductor operculi. Insertion is on the medial faces of the opercle and hyomandibula. The posterior muscle subdivision is the levator operculi, which has a tendinous origin and inserts on the posteromedial region of the opercle.

#### Calamoichthys calabaricus (not illustrated)

Facial musculature as in *Polypterus senegalus* except for the following features. The origin of the constrictor mandibularis dorsalis extends more posteriorly over the dermopterotic. The spiracularis has a tendinous origin only on that bone. The dilatator operculi is thinner at its distal portion than that of *P. senegalus*.

#### Muscle synonymy for Polypteridae

Adductor mandibulae: adductor mandibulae ([13–15], [16] (embryo), [17] (embryo), [18]); adductor mandibulae complex [19].

Adductor mandibulae, segmentum mandibulo-facialis: adductor mandibulae [20]; external portion of adductor mandibulae [21]; posterolateral division of the adductor mandibulae [18].

Adductor mandibulae, segmentum facialis: adductor mandibulae externus ([16] (adult), [17] (adult)); masseter [13–15].

Adductor mandibulae, segmentum facialis, pars ricto-malaris: A2 [19]; lateral component of posterolateral division of adductor mandibulae, AM2 [18]; superficial or upper portion (text) or division (figure) of adductor mandibulae, Am^1^ [20]; upper part of masseter [13].

Adductor mandibulae, segmentum facialis, pars stegalis: deeper or lower portion (text) or division (figure) of adductor mandibulae, Am^2^: [20]; lower part of masseter [13]; medial component of posterolateral division of the adductor mandibulae [18]; posterior subsection of A3, A3p [19].

Adductor mandibulae, segmentum buccalis: internal portion of adductor mandibulae [21]; medial division of adductor mandibulae [18].

Adductor mandibulae, segmentum buccalis, pars orbitalis: adductor mandibulae medius ([16] (adult), [17] (adult)); anterior subsection of A3, A3a [19]; temporalis [13–15, 18, 20].

Adductor mandibulae, segmentum buccalis, pars sphenoidalis: adductor mandibulae internus ([16] (adult), [17] (adult)); medial subsection of A3, A3p [19]; pterygoid [13–15]; pterygoideus [18, 20].

Adductor mandibulae, segmentum mandibularis: Aω [19]; adductor mandibulae intramandibularis ([16] (adult)); intramandibular [14]; intramandibular adductor division [18]; intramandibularis ([17] (adult)); mandibular portion (text) or division (figure) of adductor mandibulae, Am^3^ [20].

Constrictor mandibularis dorsalis: constrictor dorsalis ([17] (embryo), [19]); constrictor i dorsalis [16]; constrictor I dorsalis [14]; single primitive levator arcus palatini [20].

Constrictor mandibularis dorsalis, levator arcus palatini: levator arcus palatini ([14], [17] (adult), [18, 19]); levator arcus palatini plus protractor hyomandibularis [20]; levator maxillae superioris [13, 15].

Constrictor mandibularis dorsalis, levator arcus palatini, pars externa: levator arcus palatini [20]; superficial fibers of levator arcus palatini [14].

Constrictor mandibularis dorsalis, levator arcus palatini, pars interna: deep fibers of levator arcus palatini [14]; deeper layer of levator arcus palatini [17]; protractor hyomandibularis [20].

Constrictor mandibularis dorsalis, dilatator operculi: dilatator operculi ([14], [17] (adult), [18–21]); part of protractor hyomandibularis [13]; protractor hyomandibularis [15].

Constrictor mandibularis dorsalis, spiracularis: part of protractor hyomandibularis [13]; spiracularis [14, 17, 19, 20].

Constrictor hyoideus dorsalis: adductor hyomandibularis plus adductor operculi (considered a single muscle, labelled adductor operculi, Ao, in figures) [20]; retractor hyomandibularis et opercularis (considered as a single muscle) ([21] (adult)).

Constrictor hyoideus dorsalis, adductor hyo-operculi: adductor hyomandibulae [18, 19]; adductor hyomandibularis [15]; retractor hyomandibularis [13].

Constrictor hyoideus dorsalis, levator operculi: adductor operculi [18, 19]; levator operculi (figure only) [15]; opercularis [13].

### Acipenseriformes

#### Acipenser fulvescens (Fig. 3)

The entire adductor mandibulae is restricted to the mandibular arch, lacking any connection with the neurocranium and hyoid arch. The muscle is subdivided at origin into two main divisions. The lateralmost division apparently corresponds to a compound segmentum mandibulo-facialis. It arises from the palatopterygoid and pars autopalatina of the palatoquadrate cartilage, proceeds through the adductor fenestra, and inserts on the dentary and posterolateral region of Meckel’s cartilage. The entire mandibulo-facialis has a unique arched shape and exhibits a small but strong aponeurosis along its anterior border, at the contact area with the maxilla (= dermopalatine of some authors; see “Remarks on the upper jaw of acipenseriforms”) and pars autopalatina of the palatoquadrate cartilage. This aponeurosis is herein interpreted to be an intersegmental aponeurosis that marks the partial separation between a dorsal segmentum facialis and a ventral segmentum mandibularis (see “Homologies of the facial muscles in the Actinopterygii”). Posteriorly, fibers of the two segments are fully continuous with each other. Additionally, the segmentum facialis is partially divided into an anterolateral pars malaris and a posteromedial pars ricto-stegalis.

**Fig. 3:**
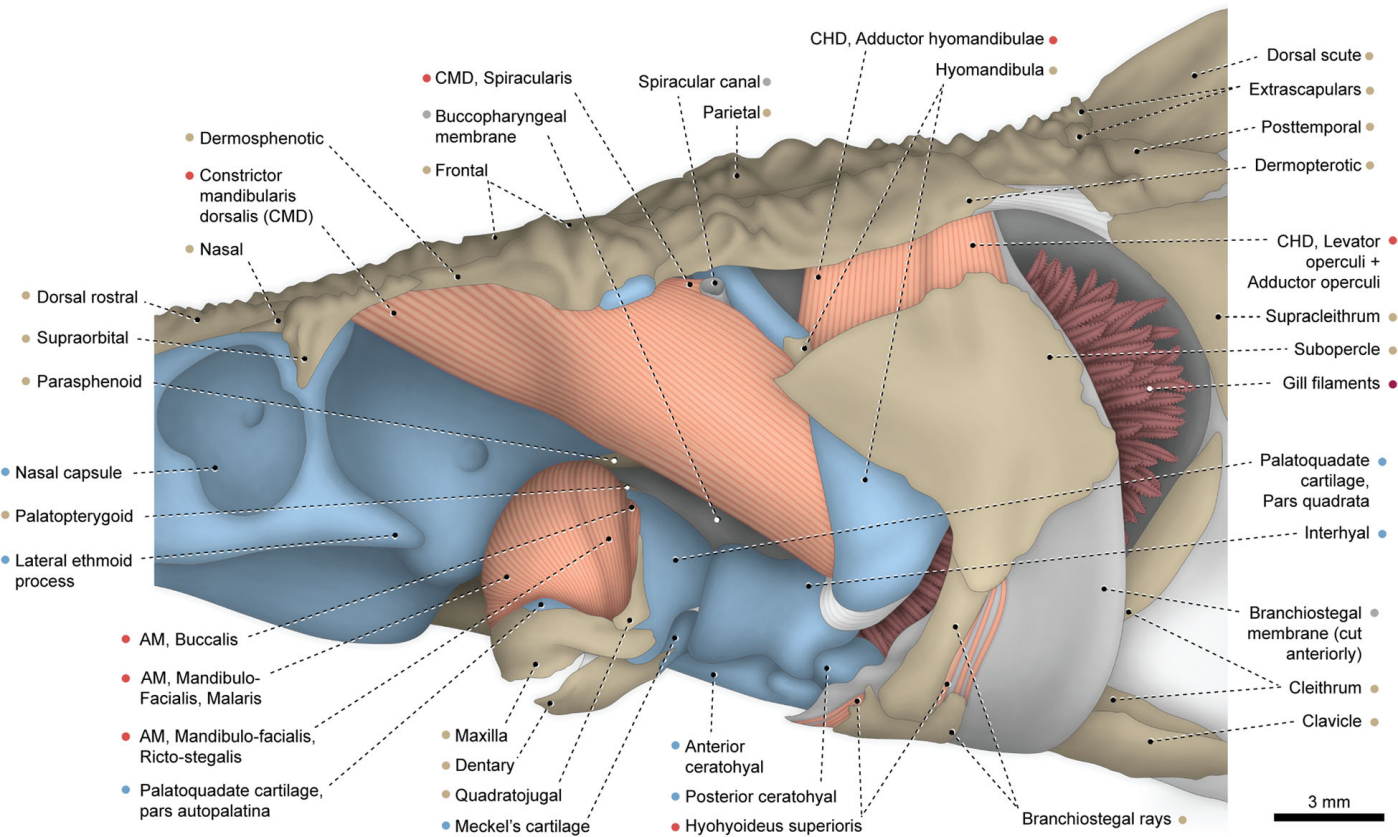
Cranial musculoskeletal system of *Acipenser fulvescens*, MZUSP 48364; left lateral view. Anteroventral rostral bones, jugal, postorbital, and anterior region of branchiostegal membrane removed. Abbreviations: AM, adductor mandibulae; CHD, constrictor hyoideus dorsalis; CMD, constrictor mandibularis dorsalis. Color code: Salmon, muscle; sand, bone; blue, cartilage; white, tendon or ligament (regular connective tissue); dark grey, irregular connective tissue; wine, gills

A small, undivided segmentum buccalis of the adductor mandibulae originates from the pars quadrata of the palatoquadrate cartilage. Insertion is on the posteromedial region of Meckel’s cartilage. Our specimens have the partes autopalatina and quadrata non-ossified into autogenous autopalatine and quadrate, respectively. However, in large specimens these bones probably serve as additional sites of origin for the segmenta mandibulo-facialis and buccalis, respectively.

The constrictor mandibularis dorsalis is a massive muscle partially divided at its origin into a lateral and a medial section. The lateral section has a wide origin on the orbital region of the cartilaginous neurocranium, dermosphenotic, and frontal. The orbitosphenoid was not ossified in our specimens, but this bone may also serve as origin site for this section in larger individuals. The medial section of the muscle arises from the region of the chondrocranium adjacent to the ascending ramus of the parasphenoid. Shortly after their origins, the lateral and medial sections merge and the constrictor mandibularis dorsalis inserts primarily on the anteromedial region of the hyomandibula. A few dorsolateral fibers correspond to the spiracularis, as they inconspicuously diverge from the main muscle mass and attach to the connective tissue forming the spiracle rim. Also, some ventromedial fibers of the constrictor mandibularis dorsalis converge to a thin but strong tendon that attaches to the posteromedial portion of the interhyal.

The constrictor hyoideus dorsalis is divided into two juxtaposed and completely separated muscles, both originating from the region of the chondrocranium located ventromedially to the dermopterotic. The anterior muscle corresponds to the adductor hyomandibulae and inserts along the posterior region of the hyomandibula. The posterior muscle inserts on the posterodorsal portion of the medial face of the subopercle and, therefore, apparently corresponds to a compound adductor operculi + levator operculi.

#### Muscle synonymy for Acipenseridae

Adductor mandibulae: adductor mandibulae [14, 15, 17, 21–23].

Adductor mandibulae, segmentum mandibulo-facialis: symphysial portion of adductor mandibulae, ams [14, 23].

Adductor mandibulae, segmentum facialis, pars malaris: superficial fibers or intermediate portion of symphysial portion of adductor mandibulae [23].

Adductor mandibulae, segmentum facialis, pars ricto-stegalis: deeper fibers or deeper portion of symphysial portion of adductor mandibulae [23].

Adductor mandibulae, segmentum buccalis: articular (caudal) portion of adductor mandibulae, ama [14, 23].

Constrictor mandibularis dorsalis: constrictor dorsalis ([17] (embryo)); constrictor I dorsalis ([14] (embryo)); constrictor mandibularis dorsalis ([23](embryo)); levator maxillae superioris ([21] (embryo)); protractor hyomandibulae ([15], [23] (adult)); protractor hyomandibularis ([14] (adult), [21] (adult), [22, 24]); protractor of hyomandibula ([17] (adult)).

Constrictor mandibularis dorsalis, lateral section: superficial portion of protractor hyomandibulae [23].

Constrictor mandibularis dorsalis, medial section: deep portion of protractor hyomandibulae [23].

Constrictor hyoideus dorsalis: dorsal hyoid constrictor [23].

Constrictor hyoideus dorsalis, adductor hyomandibulae: levator hyoidei ([21] (embryo)); retractor hyomand. [24]; retractor hyomandibulae [23]; retractor hyomandibularis ([14, 15], [21] (adult), [22]).

Constrictor hyoideus dorsalis, complex adductor operculi + levator operculi: levator operculi [15]; opercularis [21–24].

#### Polyodon spathula (Figs. 4 and 5)

Attachments of the adductor mandibulae are restricted to skeletal elements of the first pharyngeal (= mandibular) arch. Two muscle segments completely separated from each other are recognized: the facialis and buccalis. The segmentum facialis is an elongate, nearly horizontal muscle that originates from the palatopterygoid and pars autopalatina of the palatoquadrate cartilage. In its course towards insertion, the segmentum facialis bends ventrally and traverses the adductor fenestra, passing between the lateral face of the pars quadrata of the palatoquadrate cartilage and the medial face of the maxilla (= dermopalatine of some authors; see “Remarks on the upper jaw of acipenseriforms”). Near to its insertion, most fibers of the segmentum facialis associate with a strong intersegmental aponeurosis, which attaches ventrally to the medial face the dentary and to a deep groove at the posterior portion of Meckel’s cartilage. A few posteriormost fibers of the muscle segment attach directly to the foregoing groove. The segmentum mandibularis is absent.

**Fig. 4:**
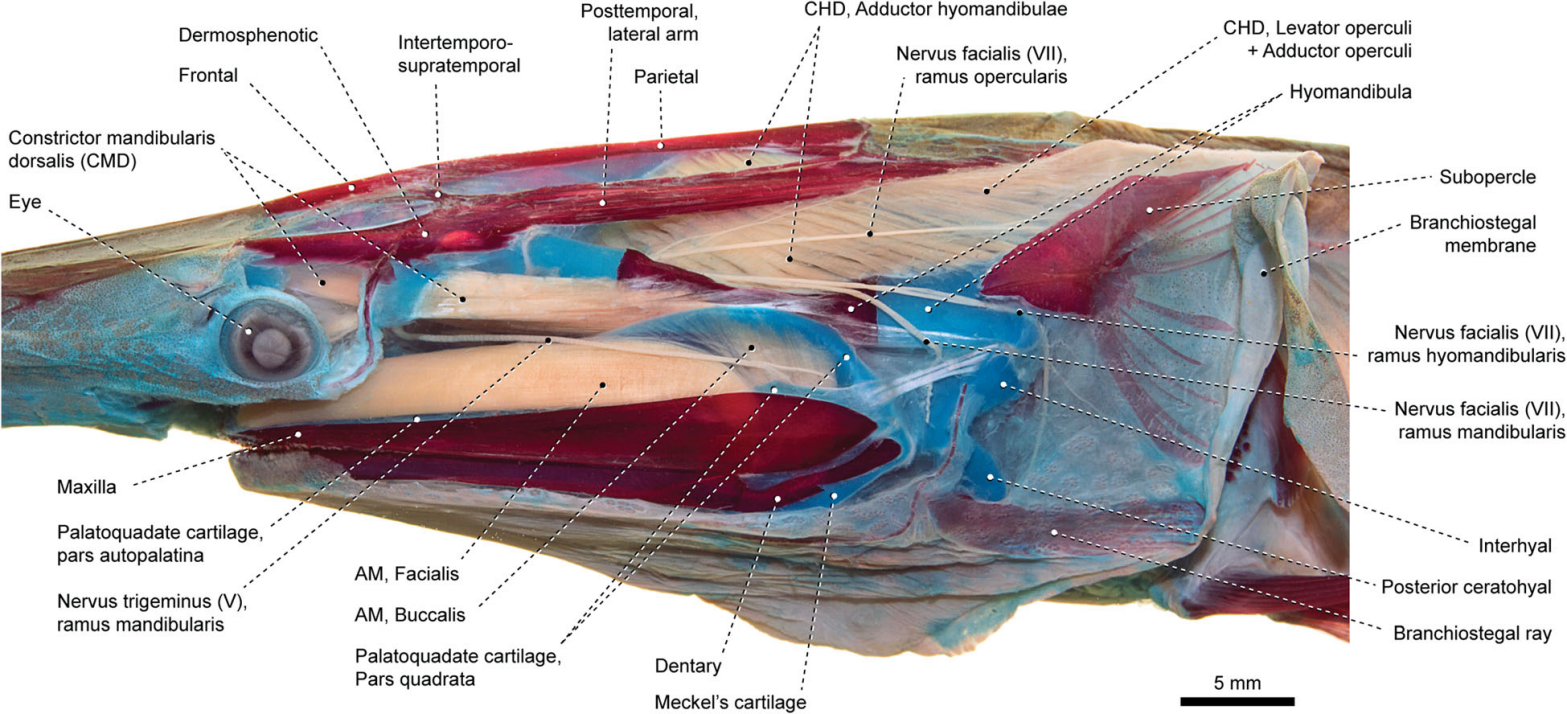
Cranial musculoskeletal system of *Polyodon spathula*, VIMS 12227; right lateral view (horizontally flipped) of partially dissected specimen stained for muscle dissection. Abbreviations: AM, adductor mandibulae; CHD, constrictor hyoideus dorsalis; CMD, constrictor mandibularis dorsalis

**Fig. 5:**
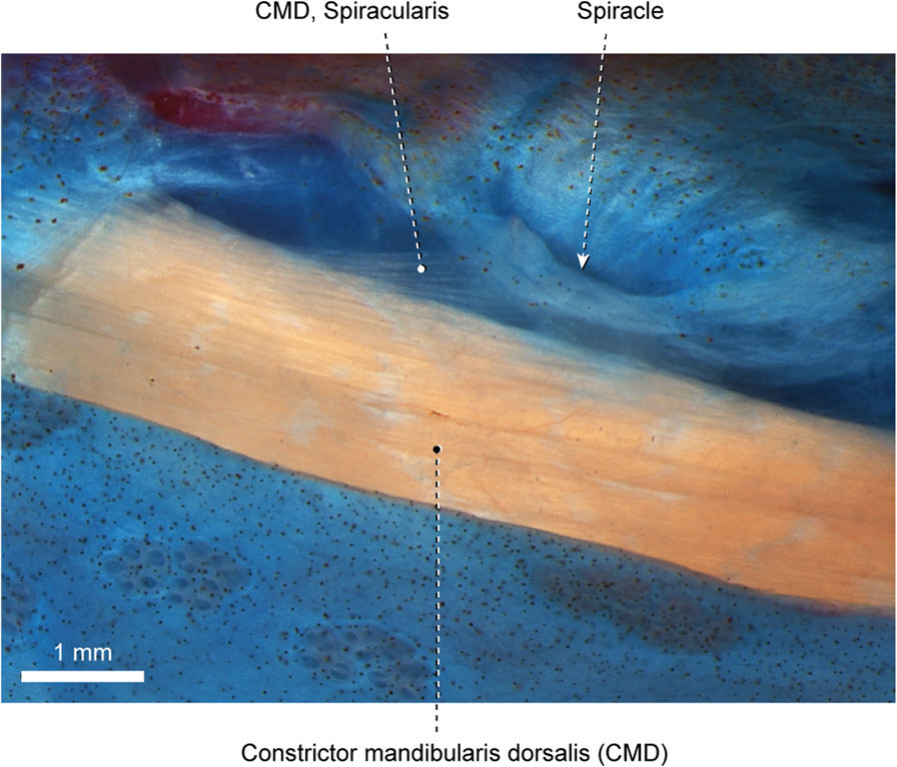
Spiracle and anterodorsal portion of constrictor mandibularis dorsalis of *Polyodon spathula*, VIMS 12227; left lateral view

A small, flat segmentum buccalis originates from the pars quadrata of the palatoquadrate cartilage and inserts on the posteromedial region of Meckel’s cartilage. The autopalatine is non-ossified in our specimen, but this ossification may be additionally involved in the origin of that muscle segment in larger specimens.

The constrictor mandibularis dorsalis is an elongate muscle completely divided at its origin into a lateral and a medial section. The lateral section is partially differentiated into an external subsection, with origin on the posterior face of the orbital region of the cartilaginous neurocranium, and an internal subsection, which arises from the lateral face of the chondrocranium lying medial to the orbit. Fibers of the medial section emerge from the region of the chondrocranium located anterior to the ascending ramus of the parasphenoid. Towards insertion, the lateral and medial sections merge to each other and almost the entire constrictor mandibularis dorsalis inserts along the anterior region of the hyomandibula. A few dorsolateral fibers, corresponding to the spiracularis, subtly attach to the connective tissue surrounding the spiracular opening (Fig. 5).

The constrictor hyoideus dorsalis is a large laminar muscle consisting of several juxtaposed parallel fibers with origin on the ventrolateral region of the chondrocranium and parietal. The muscle proceeds ventrally passing medial to the lateral arm of the posttemporal. The constrictor hyoideus dorsalis is undivided along its entirety, but a regionalization into anterior and posterior portions is noticeable. Fibers are more densely arranged on the anterior portion of the muscle, which apparently corresponds to the adductor hyomandibulae because of its insertion on the posterior region of the hyomandibula. The posteriormost fibers of the constrictor hyoideus dorsalis are thinner and more spaced from each other than those at the anterior portion of the muscle. This posterior portion of the muscle inserts on the medial face of the subopercle and, therefore, possibly corresponds to a compound adductor operculi + levator operculi.

#### Muscle synonymy for Polyodontidae

Adductor mandibulae: adductor mandibulae [14, 17]; adductor mandibularis [25].

Adductor mandibulae, segmentum facialis: adductor mandibulae 1, Ad.m.^1^ [15]; superficial part of adductor mandibularis, m.adm. [25]; symphysial portion of adductor mandibulae, ams [14].

Adductor mandibulae, segmentum buccalis: adductor mandibulae 2, Ad.m.^2^ [15]; articular (caudal) portion of adductor mandibulae, ama [14]; mesial part of adductor mandibularis, m.adm.’ [25].

Constrictor mandibularis dorsalis: constrictor I dorsalis ([14] (embryo)); protractor hyomandibularis, m.pro. ([14] (adult), [15, 25]).

Constrictor mandibularis dorsalis, lateral section: larger portion of protractor hyomandibularis [25].

Constrictor mandibularis dorsalis, medial section: smaller portion of protractor hyomandibularis [25].

Constrictor hyoideus dorsalis: primitive superficial constrictor [25].

Constrictor hyoideus dorsalis, adductor hyomandibulae: retractor hyomandibularis [14, 25]; retractor hyomandibularis et operculi [15].

Constrictor hyoideus dorsalis, complex adductor operculi + levator operculi: levator operculi [15]; opercularis [25].

### Lepisosteiformes

#### Lepisosteus platostomus (Fig. 6)

The adductor mandibulae displays a highly intricate architecture. Two major muscle segments are recognized, the facialis and buccalis; the segmentum mandibularis is absent. In the segmentum facialis, the pars stegalis is partially separated along its dorsoposterior region from the pars ricto-malaris. The latter originates primarily from the quadratojugal, palatoquadrate cartilage, hyomandibula, and preopercle. The ricto-malaris continues as a mostly undivided muscle mass along its entire extent, except for a subtle partial differentiation between the rictalis and malaris observed along their lateral faces. The pars stegalis originates from the metapterygoid, symplectic, hyopalatine membrane, quadrate, palatoquadrate cartilage, and an insertional aponeurosis at the ventral face of the levator arcus palatini. Fibers of both the stegalis and ricto-malaris converge to a large aponeurosis that accommodates part of the eyeball and inserts on the posterior region of the supraangular and coronoid process of Meckel’s cartilage. This aponeurosis is tentatively identified as the intersegmental aponeurosis. Anterolaterally, the intersegmental aponeurosis is partially continuous with a connective tissue enwrapping the coronoid process and forming a rudimentary membrane that embeds a stout ligament between the supraangular and the maxillas. Anterodorsally, the intersegmental aponeurosis exhibits a weak tendinous connection with the insertional tendons of the pars sphenoidalis (see below). Additionally, accessory laminar tendons emerge from the dorsomedial region of the intersegmental aponeurosis, run medially through the pars orbitalis externa, and anchor themselves onto the posterodorsal border of the palatoquadrate cartilage.

**Fig. 6:**
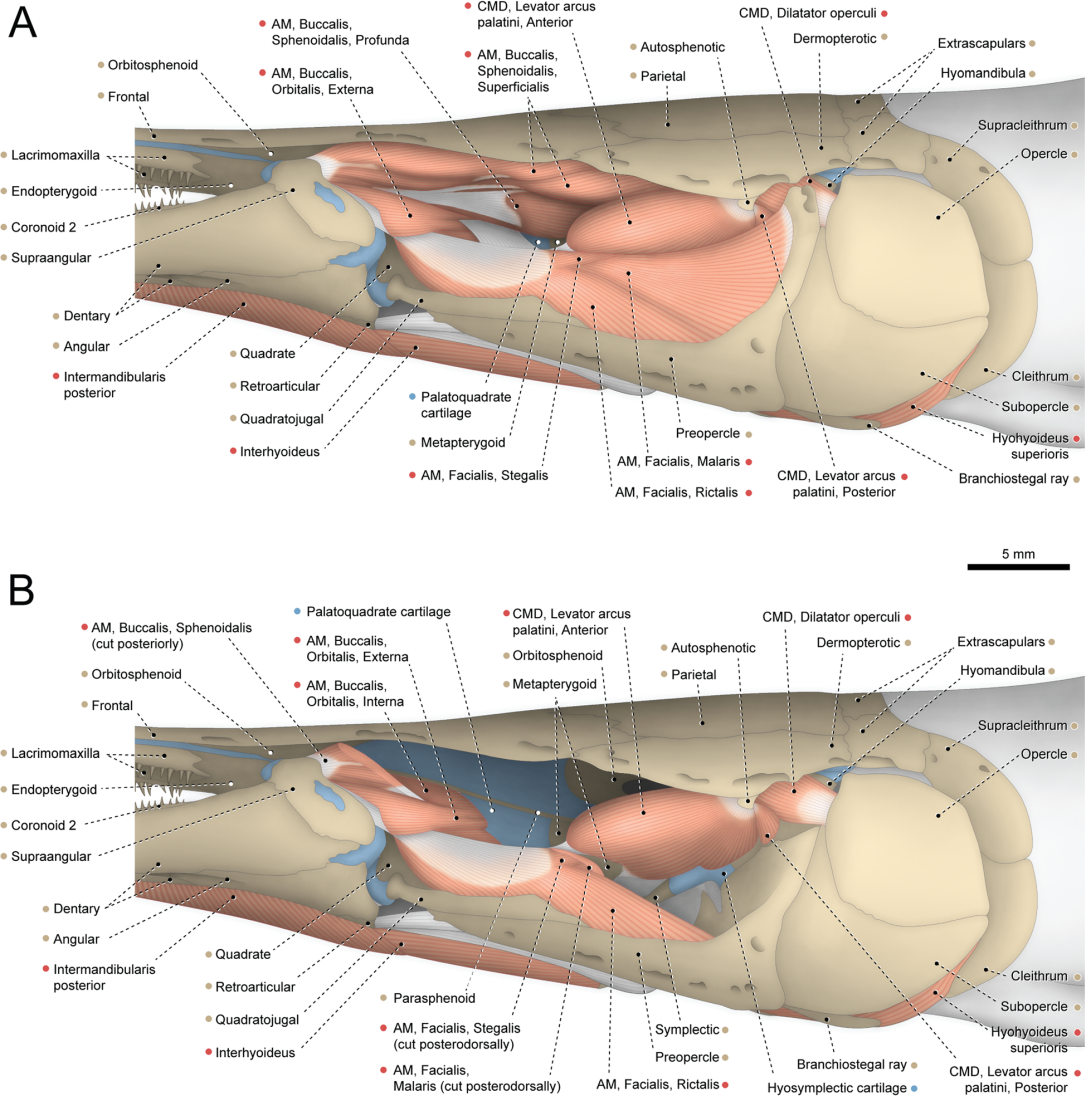
Cranial musculoskeletal system of *Lepisosteus platostomus*, USNM 54983; left lateral view; maxillary, circumorbital, and cheek bony series removed. **a** Superficial musculature. **b** Deeper musculature; dorsoposterior portions of preopercle, accessory laminar tendons of the intersegmental aponeurosis, pars sphenoidalis, pars malaris, and pars stegalis of adductor mandibulae additionally removed. Abbreviations: AM, adductor mandibulae; CMD, constrictor mandibularis dorsalis. Color code: Salmon, muscle; sand, bone; blue, cartilage; white, tendon or ligament (regular connective tissue); dark grey, irregular connective tissue

The segmentum buccalis of the adductor mandibulae exhibits well-separated partes sphenoidalis and orbitalis. The sphenoidalis is further subdivided into superficialis and profunda subsections, both arising medial to the levator arcus palatini. The pars sphenoidalis superficialis is larger and originates along the ventral surface of the frontal, dermosphenotic, and parietal. Origin of the sphenoidalis profunda is from the orbitosphenoid and, mainly, the large cartilage located just anterior to this bone. Most fibers of each sphenoidalis subsection converge to a specific strong, elongate insertional tendon. Anteriorly, the tendons serving each subsection merge to each other and attach to the medial face of the coronoid regions of Meckel’s cartilage and, most prominently, the prearticular. Fibers of anteromedial portion of the sphenoidalis superficialis do not associate with these insertional tendons and directly attach to the supraangular, prearticular, and Meckel’s cartilage.

The pars orbitalis of the segmentum buccalis lies ventral to the pars sphenoidalis and is partially continuous posteroventrally with the pars stegalis of the segmentum facialis. Origin of the pars orbitalis is primarily from the anterodorsal region of the palatoquadrate cartilage. Shortly after this origin, the insertional tendon of the pars sphenoidalis profunda (see above) traverses the orbitalis, partially dividing this section into orbitales interna and externa. Fibers of the latter subdivision attach to the dorsal surface of the intersegmental aponeurosis and the posteromedial region of the supraangular. The posterior fibers of the pars orbitalis interna converge to the insertional tendon of the sphenoidalis profunda and the intersegmental aponeurosis (see above). The anterior portion of the orbitalis interna directly inserts on the medial face of the lower jaw, at the articular and Meckel’s cartilage.

The constrictor mandibularis dorsalis is completely subdivided into two main muscles: an anterior levator arcus palatini and a posterior dilatator operculi. The levator arcus palatini is further subdivided. The pars anterior of the muscle originates primarily from the ventral faces of the autosphenotic and dermopterotic. Towards insertion, the pars anterior is partially subdivided dorsally into a lateral and a medial subsection by an intervening ventrolateral process (= basipterygoid process) of the prootic. Both subsections merge to each other ventrally and the entire pars anterior of the levator arcus palatini inserts on the hyomandibula, hyosymplectic cartilage, metapterygoid, palatoquadrate cartilage, and hyopalatine membrane. A few ventralmost fibers of the pars anterior run from the hyosymplectic cartilage to the metapterygoid. The pars posterior of the muscle originates from the postotic process of the autosphenotic and inserts on the posterolateral region of the hyomandibula. The dilatator operculi extends from the autosphenotic, dermopterotic, and hyomandibula to the anterodorsal region of the opercle.

The constrictor hyoideus dorsalis is composed of two juxtaposed but completely separated muscles. The anterior one is the adductor hyomandibulae, with origin from both the osseous and cartilaginous portions at the posteroventral region of the prootic. Insertion is on the medial aspects of the hyomandibula and hyosymplectic cartilage. The posterior division of the constrictor hyoideus dorsalis corresponds to the adductor operculi + levator operculi. This compound muscle originates from the posterior, cartilaginous region of the prootic and from a strong tendon that arises from this cartilage and associates posteriorly with some body muscles. The adductor operculi + levator operculi is mostly aponeurotic at origin and inserts on the dorsomedial regions of the opercle and opercular condyle of the hyomandibula.

#### Lepisosteus osseus (not illustrated)

Facial musculature as in *L. platostomus*, except for the following features. The connection between the lateral portion of the intersegmental aponeurosis and the ligament extending from the coronoid process to the maxillary series is more prominent. The pars orbitalis interna of the segmentum buccalis is completely separated from the pars stegalis of the segmentum facialis.

#### Muscle synonymy for Lepisosteidae

Adductor mandibulae: adductor mandibulae ([14, 15], [17] (embryo), [21] (embryo)).

Adductor mandibulae, segmentum facialis: adductor 1 [15]; adductor mandibulae ([17] (adult), [26]); external portion of adductor mandibulae ([21] (adult)); postorbitalis portion of adductor mandibulae, ampo [14].

Adductor mandibulae, segmentum buccalis: internal portion of adductor mandibulae [21].

Adductor mandibulae, segmentum buccalis, pars orbitalis: adductor 3 [15]; anterior adductor subdivision [18]; palato-mandibularis ([16] (embryo), [17] (embryo)); palatomandibularis [26].

Adductor mandibulae, segmentum buccalis, pars orbitalis interna: adductor mandibulae anterior major, amma [14]; palato-mandibularis major ([16] (adult), [17] (adult), [18]).

Adductor mandibulae, segmentum buccalis, pars orbitalis externa: adductor mandibulae anterior minor, ammi [14]; palato-mandibularis major ([16] (adult), [17] (adult), [18]).

Adductor mandibulae, segmentum buccalis, pars sphenoidalis: adductor 2 [15]; medial adductor division [18]; pars praeorbitalis of adductor mandibulae [14]; praeorbitalis ([16] (embryo), [17] (embryo), [26]).

Adductor mandibulae, segmentum buccalis, pars sphenoidalis superficialis: praeorbitalis superficialis ([14], [16] (adult), [18, 26]); superficial portion of praeorbitalis ([17] (adult)).

Adductor mandibulae, segmentum buccalis, pars sphenoidalis profunda: deep portion of praeorbitalis ([17] (adult)); praeorbitalis profundus ([14], [16] (adult), [18, 26]).

Constrictor mandibularis dorsalis: constrictor dorsalis ([17] (embryo)); constrictor i dorsalis ([16] (embryo)); constrictor I dorsalis [14]; constrictor mandibularis dorsalis [26]; levator maxillae superioris ([21] (embryo)).

Constrictor mandibularis dorsalis, levator arcus palatini: levator arcus palatini ([18], [21] (adult)); levator arcus palatini + protractor hyomandibularis [14]; protractor hyomandibularis [15].

Constrictor mandibularis dorsalis, levator arcus palatini, pars anterior: levator arcus palatini ([14], [16] (adult), [17] (adult), [26]).

Constrictor mandibularis dorsalis, levator arcus palatini, pars posterior: protractor hyomandibulae ([16] (adult), [17] (adult), [26]); protractor hyomandibularis [14].

Constrictor mandibularis dorsalis, dilatator operculi: adductor operculi [15]; dilatator operculi ([14], [16] (adult), [17] (adult), [18], [21] (adult), [26]).

Constrictor hyoideus dorsalis: constrictor hyoideus dorsalis [26].

Constrictor hyoideus dorsalis, adductor hyomandibulae: adductor arcus palatini + adductor hyomandibulae [18, 26]; adductor hyomandibularis ([21] (adult)); levator hyoidei ([21] (embryo)).

Constrictor hyoideus dorsalis, complex adductor operculi + levator operculi: adductor operculi [18, 26]; levator operculi [15]; opercularis ([21] (adult)).

### Amiiformes

#### Amia calva (Fig. 7)

Four main muscle segments are identifiable in the adductor mandibulae. The segmentum facialis is partially differentiated at origin into partes stegalis and ricto-malaris. The former originates primarily from the metapterygoid, quadrate, and posterior portion of the palatoquadrate cartilage and lies medial to the main mass of ricto-malaris. Origin of the ricto-malaris is primarily from the autosphenotic, dermopterotic, preopercle, hyomandibula, and quadrate; a few dorsolateral fibers originate from the medial face of postinfraorbital 2 (= infraorbital 5). Shortly after its origin, the ricto-malaris divides into a ventrolateral pars rictalis and a dorsomedial pars malaris. The rictalis inserts on the supraangular and a well-differentiated ligament embedded into the buccopalatal membrane. This ligament runs from the tip of the coronoid process to the anteromedial region of the maxilla and, thus, likely corresponds to an endomaxillar ligament. The malaris exhibits two partial separations along its lateral face, but such subdivisions are only superficial, with muscle fibers intermingling anteromedially. The anterolateral fibers of the malaris converge to a short tendon that inserts on the dorsal portion of the prearticular. Most fibers of this muscle section, however, are collected by a broad mandibular tendon. This tendon forms the anterolateral fold of the intersegmental aponeurosis, which has a second, posteromedial fold that corresponds to the meckelian tendon. Fibers of the stegalis attach to both the mandibular and meckelian tendons. The posterior portion of the meckelian tendon inserts on Meckel’s cartilage and, most prominently, the coronomeckelian.

**Fig. 7:**
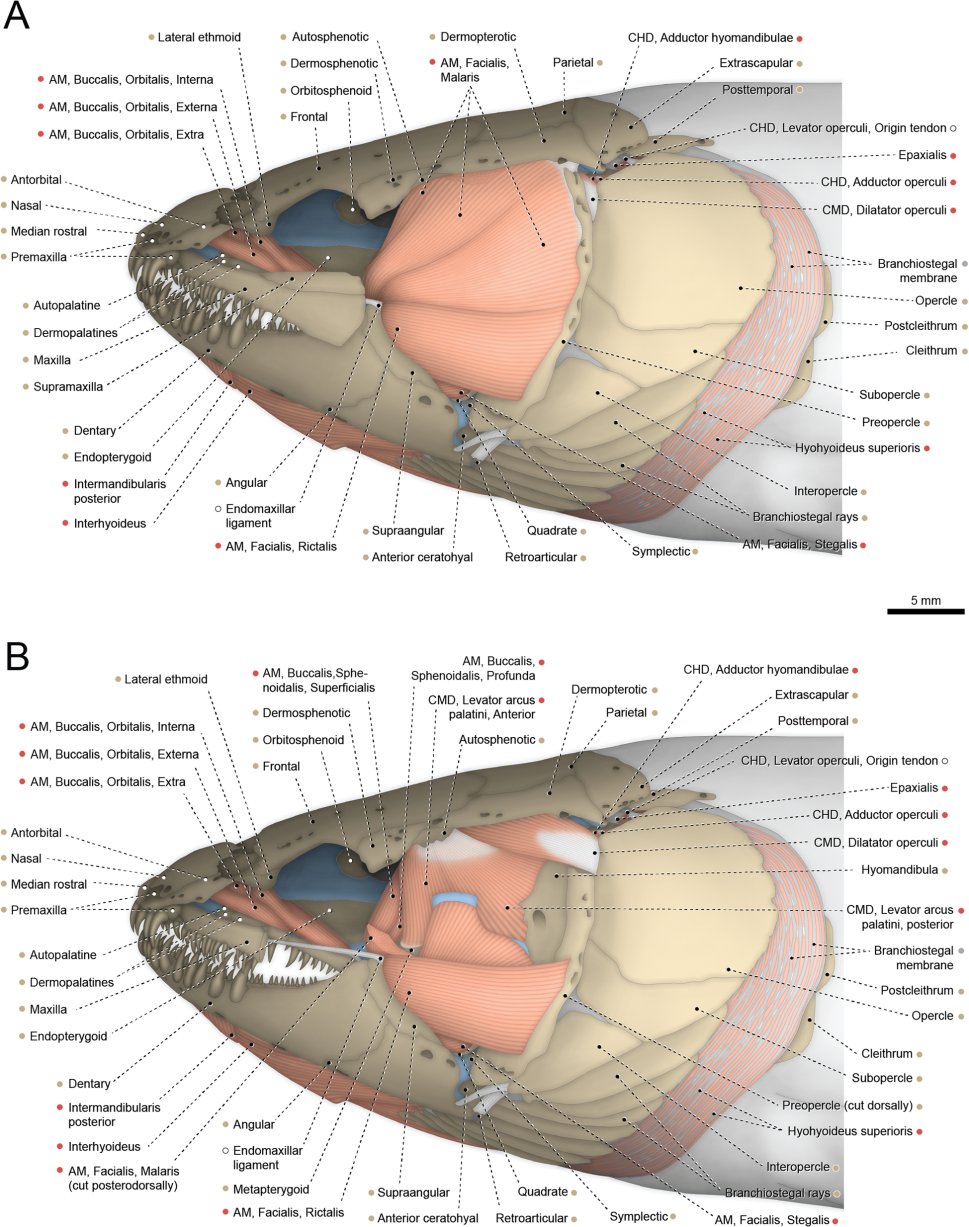
Cranial musculoskeletal system of *Amia calva*, USNM 64338; left lateral view; suborbital series and gular plate removed. **a** Superficial musculature. **b** Deeper musculature; posterior portions of maxilla and supramaxilla and dorsoposterior portions of preopercle and pars malaris of adductor mandibulae additionally removed. Abbreviations: AM, adductor mandibulae; CHD, constrictor hyoideus dorsalis; CMD, constrictor mandibularis dorsalis. Color code: Salmon, muscle; sand, bone; blue, cartilage; white, tendon or ligament (regular connective tissue); dark grey, irregular connective tissue

The segmentum mandibularis is anteriorly subdivided into an internal and an external section. This subdivision occurs in a parasagittal plane and, thus, apparently does not correspond to the typical subdivisions of the segmentum mandibularis of teleosts into a dorsal pars coronalis a ventral pars mentalis. The internal section of the segmentum mandibularis of *Amia* is smaller and extends from the anterior portion of the meckelian tendon to Meckel’s cartilage. Origin of the larger external section of the segmentum mandibularis is from the mandibular tendon; its insertion is on the medial aspects of the dentary and angular.

The segmentum buccalis of the adductor mandibulae is completely subdivided into partes sphenoidalis and orbitalis. The sphenoidalis originates medial to the levator arcus palatini, on the ascending ramus of the parasphenoid and anterodorsal border of the hyomandibula. Shortly after this origin, the section divides into two subsections. The sphenoidalis superficialis attaches to the anterodorsal region of the mandibular tendon. Fibers of the pars profunda converge to a flat tendon that merges with the insertional tendon of the pars orbitalis profunda (see below). This compound insertional tendon proceeds ventroposteriorly passing between the mandibular and meckelian tendons of the intersegmental aponeurosis. Ventrally, the meckelian tendon and the referred insertional tendon fuse to each other, but it is possible to distinguish the tendinous fibers of the latter proceeding posteroventrally until its attachment on the coronomeckelian.

The pars orbitalis of the segmentum buccalis is composed of two sections, one of which completely lost its connection with the lower jaw. The pars orbitalis externa originates from the antorbital and inserts on the lateral region of the autopalatine and anteroventral portion of the palatoquadrate cartilage. Origin of the orbitalis interna is from the lateral ethmoid, anteromedial portion of the palatoquadrate cartilage, and a strong ligament that interconnects these skeletal elements. Fibers of the section converge to an elongate tendon that merges with the insertional tendon of the sphenoidalis interna (see above). In one examined specimen, most of the referred ligament between the lateral ethmoid and palatoquadrate cartilage is replaced by muscle fibers. Due to its close proximity with the pars orbitalis, we designated this muscle the pars orbitalis extra. The presence of this tiny muscle is likely anomalous, as it is absent in the second examined specimen of *Amia* and has never been reported in the literature [14, 16–18, 24, 27].

The constrictor mandibularis dorsalis originates as an undivided muscle mass from the lateral regions of the dermosphenotic, autosphenotic, and dermopterotic, as well as from the ascending process of parasphenoid. Shortly after this common origin, the muscle subdivides in to an anterior levator arcus palatini and a posterior dilatator operculi. The latter inserts onto the anterodorsal region of the opercle. Along its lateral face, the levator arcus palatini additionally exhibits an inconspicuous partial separation into partes anterior and posterior. Such a differentiation is only superficial, with the two sections deeply merging with each other medially. Insertion of the whole muscle is on the anterodorsal region of the hyomandibula and dorsal portion of the metapterygoid.

The constrictor hyoideus dorsalis is separated at origin into an anterior adductor hyo-operculi (adductor hyomandibulae + adductor operculi) and a posterior levator operculi. The former compound muscle originates from the prootic and intercalar. The levator operculi arises from a strong and elongated tendon that attaches to the ventral face of the joint among parietal, dermopterotic, and extrascapular; part of the epaxialis also associates with this tendon. A partial separation into adductor operculi—fibers associated with the anteromedial face of the opercle—and adductor hyomandibulae—fibers attached to the posteromedial face of the hyomandibula—is observed along the lateral face of the adductor hyo-operculi. The three partially separated muscles become continuous with each other ventromedially and the entire constrictor hyoideus dorsalis inserts along the medial faces of the hyomandibula and opercle.

#### Muscle synonymy for Amiidae

Adductor mandibulae: adductor mandibulae ([16] (embryo), [17] (embryo)).

Adductor mandibulae, segmentum mandibulo-facialis: adductor mandibulae ([14, 15], [21] (embryo), [24, 27]); external portion of adductor mandibulae ([21] (adult)).

Adductor mandibulae, segmentum facialis: upper part of adductor mandibulae, A_2_ + A_3_ [14].

Adductor mandibulae, segmentum facialis, pars ricto-malaris: A_2_ [14]; pars superficialis plus pars temporalis lateralis of adductor mandibulae [15]; superficial part of adductor mandibulae, AM^1^ (including segmentum mandibularis) [24]; superficial portion of adductor mandibulae, A_2_ [27].

Adductor mandibulae, segmentum facialis, pars rictalis: A_2_ ([17] (adult)); A_2_’ [14]; adductor mandibulae externus ([16] (adult)); lower or posterior portion of A_2_, A_2_’ [27].

Adductor mandibulae, segmentum facialis, pars malaris: A_1_ ([17] (adult)); A_2_″ plus A_2_‴ [14]; middle plus upper or anterior portion of A_2_, A_2_″ + A_2_‴ [27]; adductor mandibulae medius ([16] (adult)).

Adductor mandibulae, segmentum facialis, pars stegalis: A_3_ ([14], [17] (adult)); adductor mandibulae internus ([16] (adult)); inner or deeper portion or division of adductor mandibulae, A_3_ [27]; pars temporalis medialis of adductor mandibulae [15]; second portion of adductor mandibulae, AM^2^ (including segmentum mandibularis): [24].

Adductor mandibulae, segmentum buccalis, pars orbitalis: anterior adductor division [18]; fourth plus fifth portion of levator arcus palatini, LAP^4^ + LAP^5^ [24]; levator maxillae superioris [15]; praeorbitalis ([16] (embryo), [17] (embryo)); third plus fourth divisions of levator maxillae superioris, Lms^3^ + Lms^4^ [27].

Adductor mandibulae, segmentum buccalis, pars orbitalis interna: fourth portion of levator arcus palatini, LAP^4^ [24]; part 3 of levator maxillae superioris [18]; praeorbitalis ([14], [16] (adult), [17] (adult)); third division of levator maxillae superioris, Lms^3^ [27].

Adductor mandibulae, segmentum buccalis, pars orbitalis externa: fifth portion of levator arcus palatini, LAP^5^ [24]; fourth division of levator maxillae superioris, Lms^4^ [27]; nasalis ([14], [16] (adult), [17] (adult)); part 4 of levator maxillae superioris [18].

Adductor mandibulae, segmentum buccalis, pars sphenoidalis: first, upper, or anterior plus second, lower, or posterior parts or divisions of levator maxillae superioris, Lms^1^ + Lms^2^ [27]; medial adductor division [18]; palato-mandibularis ([16] (embryo), [17] (embryo)); portio parabasalis of adductor mandibulae [14]; parabasalis ([16] (adult), [17] (adult)); second plus third portion of levator arcus palatini, LAP^2^ + LAP^3^ [24].

Adductor mandibulae, segmentum buccalis, pars sphenoidalis superficialis: first, upper, or anterior part or division of levator maxillae superioris, Lms^1^ [27]; second portion of levator arcus palatini, LAP^2^ [24].

Adductor mandibulae, segmentum buccalis, pars sphenoidalis profunda: second, lower, or posterior part or division of levator maxillae superioris, Lms^2^ [27]; third portion of levator arcus palatini, LAP^3^ [24].

Adductor mandibulae, segmentum mandibularis: intramandibular adductor division [18]; intramandibularis [17]; mandibular portion of adductor mandibulae, Aω [27]; pars intramandibularis of adductor mandibulae [15]; weaker part of adductor mandibulae, Aω [14].

Adductor mandibulae, segmentum mandibularis, pars externa: Aω’ [14]; outer portion of Aω, Aω’ [27].

Adductor mandibulae, segmentum mandibularis, pars interna: Aω" [14]; inner portion of Aω, Aω" [27].

Constrictor mandibularis dorsalis: constrictor dorsalis ([17] (embryo)); constrictor i dorsalis ([16] (embryo)); first portion of levator arcus palatini, LAP^1^: [24]; levator maxillae superioris ([21] (embryo)).

Constrictor mandibularis dorsalis, levator arcus palatini: anterior part of first portion of levator arcus palatini [24]; levator arcus palatini ([14], [16] (adult, p. 50), [17] (adult), [18], [21] (adult), [27]); levator palato-quadrati ([16] (adult, pp. 51–52)].

Constrictor mandibularis dorsalis, levator arcus palatini, pars anterior: anterior fibers of anterior part of first portion of levator arcus palatini [24]; levator arcus palatini [15]; outer portion of levator arcus palatini [27].

Constrictor mandibularis dorsalis, levator arcus palatini, pars posterior: inner portion of levator arcus palatini [27]; posterior fibers of anterior part of first portion of levator arcus palatini [24]; protractor hyomandibularis [15].

Constrictor mandibularis dorsalis, dilatator operculi: dilatator operculi ([14, 15], [16] (adult), [17] (adult), [18], [21] (adult), [27]); posterior part of first portion of levator arcus palatini [24].

Constrictor hyoideus dorsalis: adductor hyomandibularis or adductor arcus palatini (considered to represent both muscles plus adductor operculi and levator operculi) [24]; adductor hyomandibularis plus adductor operculi plus levator operculi (considered to be continuous at insertion) [27]; levator hyoidei ([21] (embryo)).

Constrictor hyoideus dorsalis, adductor hyo-operculi: adductor hyomandibulae ([18] (text)); adductor hyomandibulae plus adductor arcus palatini ([18] (figure)); adductor hyomandibularis ([21] (adult)); adductor hyomandibularis plus adductor operculi [15, 27].

Constrictor hyoideus dorsalis, levator operculi: adductor plus levator operculi ([21] (adult)); adductor operculi plus levator operculi [18]; levator operculi [15, 27].

### Elopiformes

#### Elops lacerta (Figs. 8 and 9)

The adductor mandibulae exhibits only the facial and mandibular segments; the segmentum buccalis is absent. The segmentum facialis lacks any obvious subdivisions along its entirety and originates from the preopercle, quadrate, metapterygoid, symplectic, and hyomandibula. The ventromedial fibers of the segmentum facialis, which presumably correspond to the stegalis, converge to a flat meckelian tendon that attaches to the coronomeckelian. The remaining muscle fibers, which correspond to the ricto-malaris, insert on the mandibular tendon and retrojugal lamina, which are continuous with each other.

**Fig. 8:**
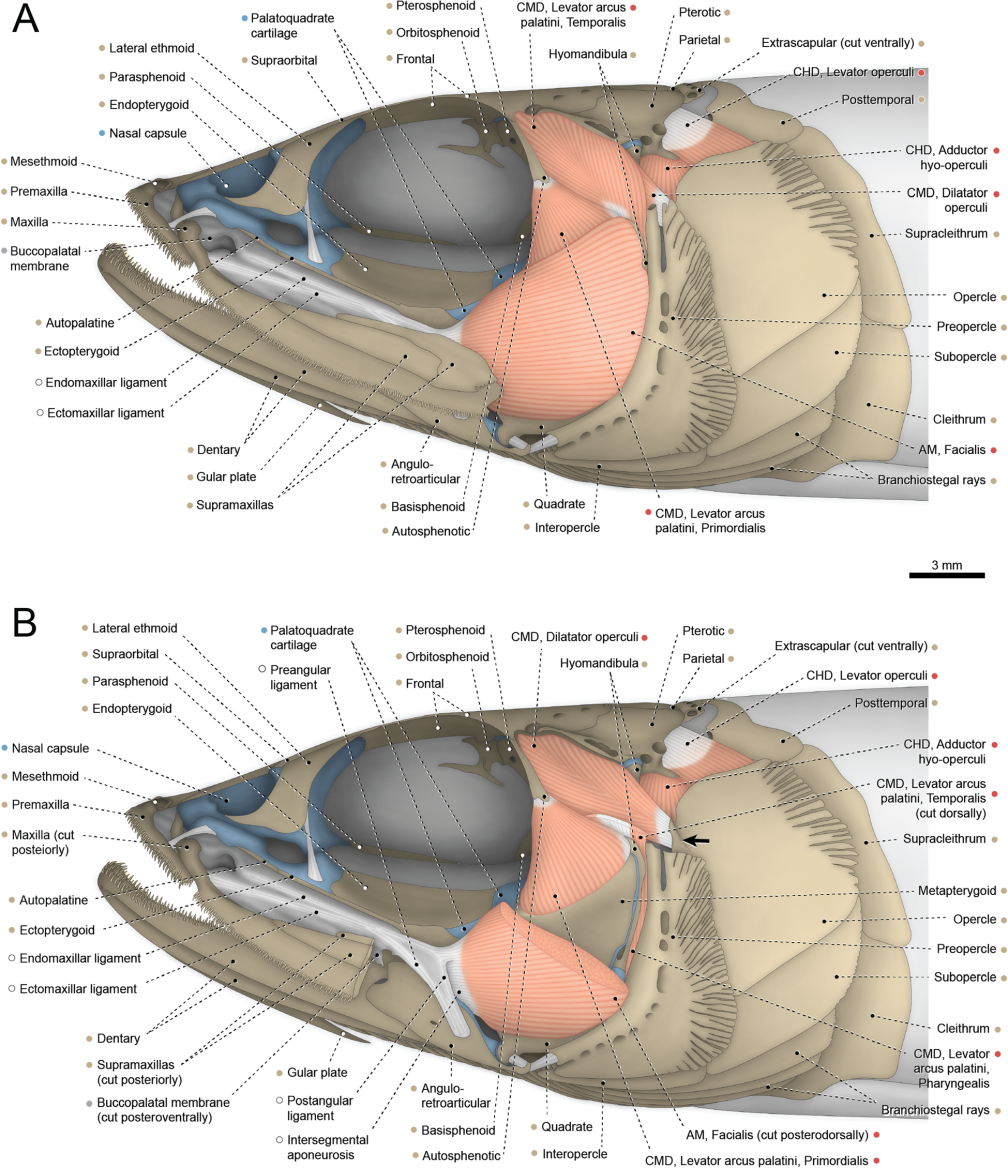
Cranial musculoskeletal system of *Elops lacerta*, MZUSP 84787; left lateral view; nasal, antorbital, infraorbital series, and ventral portion of extrascapular removed. **a** Superficial musculature. **b** Deeper musculature; posterior portions of maxilla and supramaxillas, dorsal portion of pars temporalis of levator arcus palatini, and dorsoposterior portions of preopercle and segmentum facialis of adductor mandibulae additionally removed. Arrow indicates dilatator process of opercle. Abbreviations: AM, adductor mandibulae; CHD, constrictor hyoideus dorsalis; CMD, constrictor mandibularis dorsalis. Color code: Salmon, muscle; sand, bone; blue, cartilage; white, tendon or ligament (regular connective tissue); dark grey, irregular connective tissue

**Fig. 9:**
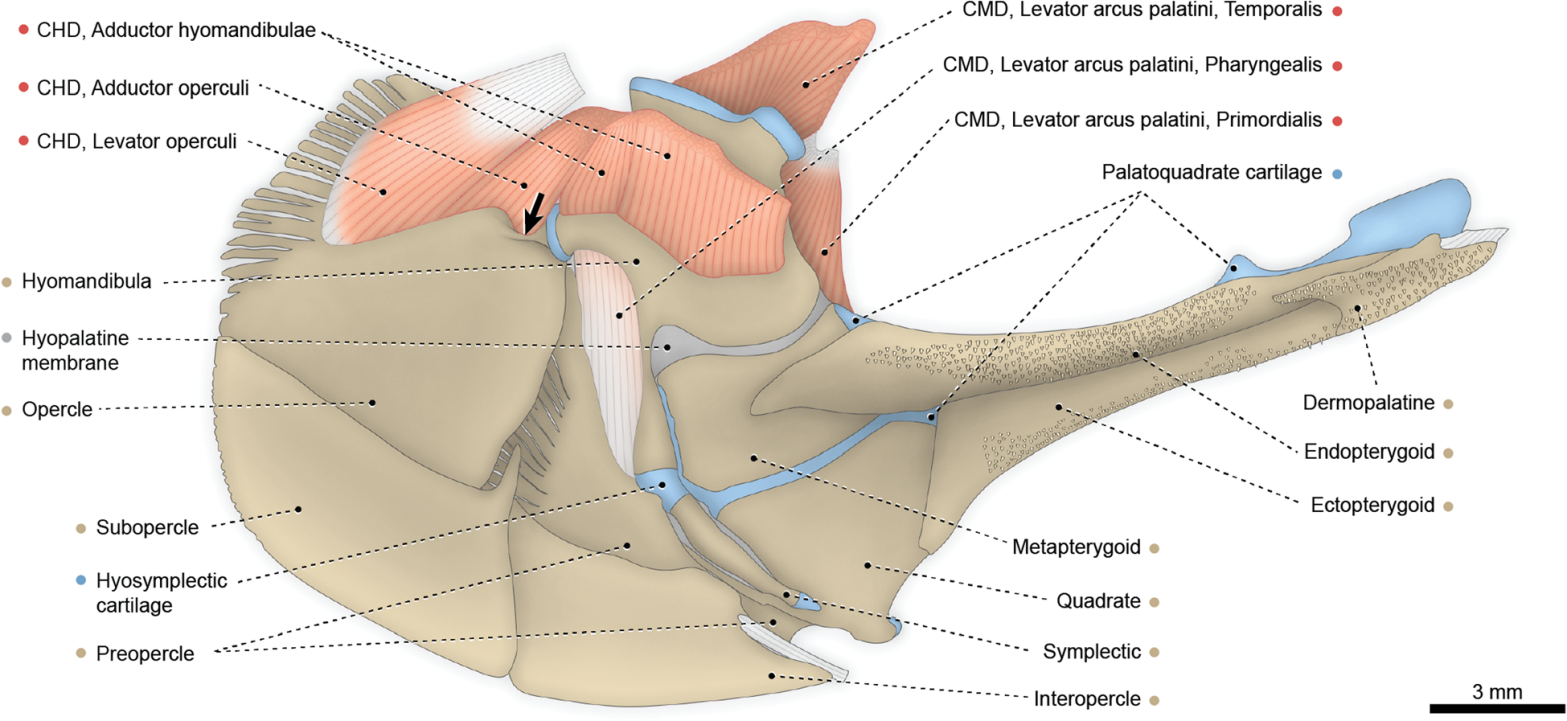
Hyopalatine arch, opercular series, and associated constrictor hyoideus dorsalis and levator arcus palatini of *Elops lacerta*, MZUSP 84787; left medial view. Arrow indicates adductor crest of opercle. Abbreviation: CHD, constrictor hyoideus dorsalis; CMD, constrictor mandibularis dorsalis. Color code: Salmon, muscle; sand, bone; blue, cartilage; white, tendon or ligament (regular connective tissue); dark grey, irregular connective tissue

The segmentum mandibularis of the adductor mandibulae is undivided and originates from the flat mandibular tendon. Insertion is on the dentary and angulo-retroarticular.

The constrictor mandibularis dorsalis is completely separated into dilatator operculi and levator arcus palatini. The later muscle has a main bulk comparable to the levator arcus palatini of other actinopterygians. This muscle portion is accordingly termed pars primordialis. Origin of this pars is mainly from the postorbital process of the autosphenotic and insertion is on the hyomandibula and metapterygoid. Two additional sections of the levator arcus palatini are present and apparently correspond to expansions of the primitive muscle (pars primordialis; see “Homologies of the facial muscles in the Actinopterygii”). These additional sections are termed partes temporalis and pharyngealis. They are partially continuous with, but clearly differentiable from, the pars primordialis. The pars temporalis is located dorsoposterior to the pars primordialis and has a nearly bipennate architecture. Fibers of the pars temporalis arise primarily from the borders of the dilatator fossa on the autosphenotic and pterotic, overlying the dilatator operculi. Insertion of that pars is on the dorsolateral border of the hyomandibula and a connective tissue between this bone and the preopercle. Muscle fibers at the contact area between the partes primordialis and temporalis of the levator arcus palatini converge ventrally into a strong tendon. Part of this tendon attaches to the hyomandibula and part continues ventrally, passing medially to the dorsal end of the preopercle and serving as the origin site for the pars pharyngealis of the levator arcus palatini. The pars pharyngealis is located at the internal face of the hyopalatal arch and exhibits a broad medial aponeurosis. Insertion is on the descending ramus of the hyomandibula and vertical ramus of the preopercle.

The dilatator operculi is nearly bipennate and almost completely covered laterally by the pars temporalis of the levator arcus palatini. Origin of the dilatator operculi is form the hyomandibula and the depression of the dilatator fossa on the autosphenotic and pterotic. The muscle inserts tendinously on an anterodorsal process of the opercle (= dilatator process).

The constrictor hyoideus dorsalis is divided at origin into two muscles. The adductor hyo-operculi (adductor hyomandibulae + adductor operculi) originates musculously from the pterotic, prootic, and intercalar. The levator operculi arises from an aponeurosis attached to the posterior border of the pterotic. Shortly after these origins the two muscles merge to each other and the whole constrictor hyoideus dorsalis inserts along the medial faces of the opercle and hyomandibula. In addition to the partial separation at origin between levator operculi and adductor hyo-operculi, the constrictor hyoideus dorsalis exhibits other superficial separations. Such divisions are incomplete, with fibers intermingling with each other in deeper portions of the muscle. One of these partial separations is between the muscle bundles corresponding to the adductor operculi, attached to the anterior region of the opercle, and those corresponding to the adductor hyomandibulae, inserting on the hyomandibula. A second partial separation is observed only on the medial face of the muscle, between a larger anterior and a smaller posterior section of the adductor hyomandibulae.

#### Muscle synonymy for Elopidae

Adductor mandibulae: adductor mandibulae [1, 6, 28].

Adductor mandibulae, segmentum facialis: adductor mandibulae [29]; section A2 [1]; segmentum facialis [6, 28].

Adductor mandibulae, segmentum mandibularis: intramandibularis [29]; section Aw [1]; segmentum mandibularis [6, 28].

Constrictor mandibularis dorsalis: constrictor dorsalis [1].

Constrictor mandibularis dorsalis, levator arcus palatini: levator arcus palatini [1].

Constrictor mandibularis dorsalis, levator arcus palatini, pars temporo-primordialis: levator arcus palatini [29].

Constrictor mandibularis dorsalis, levator arcus palatini, pars primordialis: levator arcus palatini ([6] (figure)).

Constrictor mandibularis dorsalis, levator arcus palatini, pars temporalis: dilatator operculi ([6] (figure)).

Constrictor mandibularis dorsalis, dilatator operculi: dilatator operculi [1, 29].

Constrictor hyoideus dorsalis: adductor arcus palatini plus adductor operculi plus levator operculi ([1] (figure)); constrictor hyoideus dorsalis ([1] (text)].

Constrictor hyoideus dorsalis, adductor hyomandibulae [29].

Constrictor hyoideus dorsalis, complex adductor operculi + levator operculi: adductor operculi and levator operculi (described as continuous) ([1] (text)); levator et adductor operculi [29].

Constrictor hyoideus dorsalis, adductor operculi: adductor operculi ([1] (text), [29]).

Constrictor hyoideus dorsalis, levator operculi: levator operculi ([1] (text), [29]).

#### Megalops cyprinoides (not illustrated)

Facial musculature comparable to that of *Elops lacerta* except for the following features. The ricto-malaris and stegalis of *M. cyprinoides* are better differentiated from each other anteriorly. The ventrolateral set of fibers, which corresponds to the rictalis, is more intimately associated with a well-defined preangulo-paramaxillar ligament. The tendons of the intersegmental aponeurosis are more obviously separated from each other and an accessory tendon is present. This accessory tendon passes lateral to the meckelian tendon and inserts on the angulo-retroarticular and posterior tip of the coronomeckelian bone. The pars pharyngealis of the levator arcus palatini is absent. The constrictor hyoideus dorsalis is divided at origin into an anterior adductor hyomandibulae and a posterior complex formed by the adductor operculi plus levator operculi. The adductor hyomandibulae further subdivides ventrally into anterior and posterior sections. The adductor operculi and levator operculi are partially separated from each other on both their lateral and medial faces.

#### Muscle synonymy for Megalopidae

Adductor mandibulae: adductor mandibulae [6].

Adductor mandibulae, segmentum facialis: adductor mandibulae [29]; segmentum facialis [6].

Adductor mandibulae, segmentum facialis, pars ricto-malaris: A_1_A_2_ [29]; pars ricto-malaris [6].

Adductor mandibulae, segmentum facialis, pars stegalis: A_3_ [29]; pars stegalis [6].

Adductor mandibulae, segmentum mandibularis: intramandibularis [29]; segmentum mandibularis [6].

Constrictor mandibularis dorsalis, levator arcus palatini, pars temporo-primordialis: levator arcus palatini [29].

Constrictor mandibularis dorsalis, dilatator operculi: dilatator operculi [29].

Constrictor hyoideus dorsalis, adductor operculi: adductor operculi [29].

Constrictor hyoideus dorsalis, levator operculi: levator operculi [29].

### Hiodontiformes

#### *Hiodon tergisus* (see illustrations in Datovo & Vari [6, 28])

The adductor mandibulae is composed only of the segmenta facialis and mandibularis; the segmentum buccalis is absent. In the segmentum facialis, the partes ricto-malaris and stegalis are clearly distinguishable, but not separated, from each other at their origins. Fibers of the ricto-malaris extend more posteriorly over the suspensorium than those of the stegalis, originating from the posterior region of the hyomandibula, preopercle, symplectic, and quadrate. Fibers of the stegalis, in turn, have a distinct anterior origin on the anterodorsal part of the hyomandibula and metapterygoid. Fibers of the ricto-malaris and stegalis are largely continuous with each other along most of their lengths. Proximate to its insertion, the ricto-malaris undergoes a tenuous subdivision. A ventrolateral set of fibers, corresponding to the rictalis, passes lateral to the ramus mandibularis trigeminus nerve and inserts on the posterior region of the preangular ligament. The dorsolateral fibers corresponding to the malaris run medial to the ramus mandibularis trigeminus nerve and attach to the dentary and mandibular tendon (see below). The fibers of the stegalis converge to the medial portions of the intersegmental aponeurosis. Within this aponeurosis, the mandibular and meckelian tendons, albeit poorly differentiated, are still discernible from each other. The meckelian tendon, onto which most of the fibers of the pars stegalis attach, is flat along its medial surface and attaches to the coronomeckelian. The mandibular tendon, on the other hand, has a characteristic undulated surface texture and receives the muscle fibers of the malaris and a few fibers from the stegalis.

Anteriorly, the mandibular tendon serves as the site of origin of a small, undivided segmentum mandibularis of the adductor mandibulae. Insertion is onto the medial surface of the dentary.

The constrictor mandibularis dorsalis is completely separated into levator arcus palatini and dilatator operculi, except for a small tendinous connection at the medial region of these muscles. The levator arcus palatini originates tendinously from the postorbital process of the autosphenotic and partially subdivides ventrally into partes anterior and posterior. Insertion of the pars anterior is along the anterior region of the hyomandibula and the portion of the palatoquadrate cartilage located between that bone and the metapterygoid. The pars posterior of the levator arcus palatini inserts on the dorsoposterior region of the hyomandibula. The dilatator operculi originates from the dilatator fossa of the autosphenotic and pterotic and inserts via elongate tendon on the anterodorsal process of the opercle (= dilatator process).

The constrictor hyoideus dorsalis is divided into three main muscles: adductor hyomandibulae, adductor operculi, and levator operculi. A large gap separates the adductor hyomandibulae from the remaining components of the constrictor hyoideus dorsalis. Origin of the adductor hyomandibulae is from the parasphenoid and prootic; insertion is on the hyomandibula, metapterygoid, and endopterygoid. The adductor operculi and levator operculi are confluent at both origin and insertion, but partially separated from each other between these attachments. The two muscles originate conjointly from a broad tendinous sheet anchored primarily to the intercalar and posttemporal. Insertion of the complex adductor operculi + levator operculi in on the dorsomedial aspects of the opercle and opercular condyle of the hyomandibula.

#### *Hiodon alosoides* (not illustrated)

Facial musculature as in *Hiodon tergisus* except for the following features. The pars rictalis is better separated from the ricto-malaris. Distinction between the mandibular and meckelian tendons is much more tenuous and perceptible only due to their distinct attachment sites. The levator arcus palatini and dilatator operculi are completely separated from each other, lacking a deep tendinous connection.

#### Muscle synonymy for Hiodontidae

Adductor mandibulae: adductor mandibulae [6, 28].

Adductor mandibulae, segmentum facialis: segmentum facialis [6, 28].

Adductor mandibulae, segmentum facialis, pars ricto-malaris: pars ricto-malaris [6].

Adductor mandibulae, segmentum facialis, pars rictalis: pars rictalis [6, 28].

Adductor mandibulae, segmentum facialis, pars malaris: pars malaris [6, 28].

Adductor mandibulae, segmentum facialis, pars stegalis: pars stegalis [6, 28].

Adductor mandibulae, segmentum mandibularis: segmentum mandibularis [6, 28].

Constrictor mandibularis dorsalis, levator arcus palatini: levator arcus palatini [6].

Constrictor mandibularis dorsalis, dilatator operculi: dilatator operculi [6].

Constrictor hyoideus dorsalis, adductor hyomandibulae: adductor arcus palatini [6].

## Discussion

### Homologies of the facial muscles in the Actinopterygii

Most previous studies on the myology of basal actinopterygians focused on only one or a few closely related taxa, with little or no concern about muscle homologies across higher taxonomic levels [13, 19, 20, 22–27, 30, 31]. The few attempts to elucidate these questions presented either unclear criteria for the proposed hypotheses of homologies (= primary homologies) or relied upon one or a few attributes (e.g. muscle insertion) as landmarks for such inferences [14, 16, 18]. These approaches have produced many terminological inconsistencies and a proliferation of different names attributed to the facial muscles (see synonymies in “Results”). In order to achieve more robust hypotheses of homology that minimize the conflicts among distinct morphological attributes, we evaluated multiple features—origin, insertion, position, shape, innervation, relationship with surrounding structures—along with ontogenetic evidence from the literature [16, 17, 19, 23, 26] in a broad range of actinopterygian taxa (Table 1 and comparative material listed in Datovo & Vari [6, 28]). As a consequence, we identify relatively stable features that characterize the major subdivisions of the facial muscles in basal actinopterygians. These findings are detailed in the following paragraphs and the main properties of each muscle component are synthesized in Table 2.

**Table 2:** Main subdivisions of facial musculature in Actinopterygii

Muscle	Subdivision	Additional features
MANDIBULAR MUSCLE PRIMORDIUM		
1. Constrictor mandibularis dorsalis	1. Dorsomedial	Origin on lateral region of otic-sphenoidal portion of neurocranium; insertion on lateral face of palatoquadrate, hyomandibula, and opercle (except acipenseriforms)
1.1. Levator arcus palatini	1.1. Anterior	Insertion on lateral face of suspensorium, usually on hyomandibula and metapterygoid
1.2. Dilatator operculi	1.2. Middle	Insertion on anterodorsal region of opercle
1.3. Spiracularis	1.3. Posterior	Insertion on spiracular canal or ossicles; lost or undifferentiated from dilatator operculi in neopterygians
2. Adductor mandibulae	2. Ventrolateral	Origin on lateral region of palatoquadrate and, often, hyomandibula and neurocranium; insertion on lower jaw; additional attachments to maxilla in *Amia* and several teleosts
2.1. Segmentum facialis	2.1. Dorsolateral	Larger and most superficial division on cheek
2.1.1. Pars rictalis	2.1.1. Ventrolateral	Origin on ventroposterior region of suspensorium, usually involving quadrate
2.1.2. Pars malaris	2.1.2. Dorsolateral	Origin on dorsoposterior region of suspensorium, usually involving hyomandibula
2.1.3. Pars stegalis	2.1.3. Medial	Shorter fibers with origin on anterior region of suspensorium, usually involving metapterygoid
2.2. Segmentum buccalis	2.2. Dorsomedial	Deeper division on cheek; lost in teleosts
2.2.1. Pars orbitalis	2.2.1. Anterior	Located just posterior or anteroventral to eyeball
2.2.2. Pars sphenoidalis	2.2.2. Posterior	Origin medial to levator arcus palatini
2.3. Segmentum mandibularis	2.3. Ventral	Fully or mostly concealed laterally by lower jaw
HYOID MUSCLE PRIMORDIUM		
3. Constrictor hyoideus dorsalis	3. Dorsal	Origin on lateral region of otic-temporal region of neurocranium; insertion on medial faces of palatoquadrate, hyomandibula, and opercle
3.1. Adductor hyomandibulae	3.1. Anterior	Insertion on hyomandibula and, often, metapterygoid and endopterygoid
3.2. Adductor operculi	3.2. Middle	Insertion on the anterior region of opercle, near its articulation with hyomandibula; associated with medial crest of opercle in teleosts.
3.3. Levator operculi	3.3. Posterior	Narrow origin via elongate tendon/aponeurosis; broader insertion on posterior portion of opercle

In actinopterygians the muscles primarily associated with the mandibular arch derive ontogenetically from the mandibular muscle primordium (= mandibular muscle plate) and are innervated by the nervus trigeminus (cranial nerve V; Fig. 4) [16, 17, 26, 32]. During early development, this primordium fragments into a dorsal constrictor mandibularis dorsalis (= constrictor I dorsalis [14, 16, 33] or, simply, constrictor dorsalis [1, 17, 19]), a middle adductor mandibulae, and a ventral intermandibularis [16, 17, 26]. The two former compose the masticatory muscle plate [16, 17] and give rise to most facial muscles of adult actinopterygians. The third component of the actinopterygian facial musculature is the constrictor hyoideus dorsalis (= constrictor II dorsalis [33]), the dorsalmost element of the hyoid muscle primordium, which is innervated by the nervus facialis (cranial nerve VII; Fig. 4) [16, 17, 26, 32]. In later developmental stages, the three main muscle primordia that form the facial musculature differentiate into the several subdivisions typically found in adults: (1) the adductor mandibulae primordium gives rise to up to three main segments (segmenta facialis, buccalis, and mandibularis) and its several subdivisions (sections or partes); (2) the constrictor mandibularis dorsalis differentiates into the spiracularis, dilatator operculi, and levator arcus palatini; and (3) the constrictor hyoideus dorsalis into the adductor hyomandibulae, adductor operculi, and levator operculi (Table 2). Most interestingly, this ontogenetic pattern roughly reflects the evolutionary history of these muscles. Generalized cartilaginous fishes exhibit a poorly subdivided adductor mandibulae and mostly or fully undivided constrictor mandibularis dorsalis and constrictor hyoideus dorsalis [17, 21, 32, 34–37]. In Osteichthyes and Actinopterygii, these facial muscles differentiate into several subunits (see “Phylogenetic inferences”) but retain the primitive innervation pattern of their respective muscle primordia. That is, the trigeminal nerve (V) serves the muscle subdivisions derived from the mandibular plate (adductor mandibulae and constrictor mandibularis dorsalis) and the facial nerve (VII) those derived from the hyoid muscle plate (constrictor hyoideus dorsalis).

Basal actinopterygians have the adductor mandibulae connecting the lower jaw with the hyopalatine arch and often the neurocranium and preopercle. In polypteriforms, lepisosteiforms, and amiiforms, the segmentum facialis (sensu Datovo & Vari [6, 28]) is the largest and lateralmost component of the adductor mandibulae (Figs. 1, 6, 7 and 8). At least the external portion of this muscle segment is connected ventrally via an intersegmental aponeurosis with the segmentum mandibularis [6, 28], which invariably attaches to the dentary and other elements of the lower jaw. The largest division of the adductor mandibulae in *Acipenser* is also lateral to the remainder of the muscle and exhibits a small anterolateral intermediate aponeurosis and a ventral attachment to the dentary and Meckel’s cartilage (Fig. 3). This suggests the lateralmost division of the acipenserid adductor mandibulae corresponds to a compound segmentum mandibulo-facialis, with an intersegmental aponeurosis restricted to the anterior portion of the muscle. Such a configuration is similar to that of some generalized sharks [32, 34–37], but rare (possibly unique) among bony fishes. An intersegmental aponeurosis restricted to the anterior profile of the lateral division of the adductor mandibulae is also found in *Polyodon*, but in this taxon the aponeurosis directly attaches to the lower jaw. This lateralmost component of the adductor mandibulae is thus interpreted to correspond to the segmentum facialis only (Fig. 4), and the segmentum mandibularis in *Polyodon* presumably has been lost. Among the herein examined basal actinopterygians, the segmentum mandibularis is also absent in *Lepisosteus*.

Datovo & Vari [6, 28] identified three main sections in the segmentum facialis of teleosts: a ventrolateral pars rictalis, a dorsolateral pars malaris, and a medial pars stegalis. These partes exhibit varying degrees of differentiation from each other, but are present in virtually all teleosts. The same sections, with comparable varying degrees of differentiation, are also recognized in the segmentum facialis of basal actinopterygians. In polypteriforms, the segmentum facialis is primarily differentiated into ricto-malaris and stegalis (Fig. 1); in *Acipenser*, into malaris and ricto-stegalis (Fig. 3); in *Polyodon*, there are no subdivisions (Fig. 4); and in holosts, all three partes are identifiable (Figs. 6 and 7).

All non-teleost actinopterygians have an additional segment of the adductor mandibulae attached to the lower jaw and located medial to the segmentum facialis (Figs. 1, 3, 4, 6 and 7). This muscle segment is herein termed segmentum buccalis, in allusion to its close proximity to the buccal cavity and in opposition to the external segmentum facialis (closer to the cheek surface). Coelacanths [38, 39], lungfishes [14, 17, 40–46], and at least some generalized chondrichthyans [32, 34] exhibit an internal division of the adductor mandibulae comparable to the segmentum buccalis. Therefore, the presence of a segmentum buccalis of the adductor mandibulae is plesiomorphic for ray-finned fishes. In most basal actinopterygians, the segmentum buccalis is further subdivided into two main sections (Figs. 1, 6 and 7). The posterior one is the pars sphenoidalis, which originates from the sphenoidal region of the neurocranium, medial to the constrictor mandibularis dorsalis. The anterior subdivision of the segmentum buccalis is the pars orbitalis, located at least partially ventral to the eyeball and with insertion entirely or mostly medial to that of the pars sphenoidalis. Origin of the pars orbitalis is from the neurocranium (Polypteriformes; Fig. 1), palatoquadrate (Lepisosteiformes; Fig. 6), or both (Amiiformes; Fig. 7). In holosts the partes orbitalis and sphenoidalis are further subdivided into lateral and medial subsections (Figs. 6 and 7). Acipenseriformes are unique among actinopterygians in having a segmentum buccalis completely undivided and with origin restricted to the palatoquadrate (Figs. 3 and 4). Generalized teleosts lack any subdivision of the adductor mandibulae comparable to the segmentum buccalis (Fig. 8) [6, 28], which was thus presumably lost in the Teleostei (see Character 1).

The hypotheses of homologies herein advanced for the subdivisions of the adductor mandibulae are also consistent with the ontogenetic data reported in the literature [16, 17, 19, 23, 26]. In all basal actinopterygians, the earlier differentiation observed during the development of the adductor mandibulae is between the segmenta facialis and buccalis. Subsequently, the partes sphenoidalis and orbitalis begin to differentiate from the single, primordial segmentum buccalis, despite the enormous morphological divergences of these muscle sections in adults of the different examined taxa (compare Figs. 1, 6 and 7). Within the segmentum facialis, the stegalis is typically the first section to differentiate. The segmentum mandibularis, in turn, is the last division of the adductor mandibulae to appear during development.

The constrictor mandibularis dorsalis originates from the lateral region of the sphenoidal portion of the neurocranium (Figs. 1, 3, 4, 6, 7 and 8). At insertion, the muscle attaches to the anterolateral region of the hyomandibula and, except in acipenseriforms, some elements of the palatoquadrate. All non-acipenseriform actinopterygians have the main bulk of the constrictor mandibularis dorsalis differentiated into at least two well-defined subunits: a dilatator operculi, with insertion on the opercle, and a levator arcus palatini, with insertion on the hyomandibula and elements of the palatoquadrate (Figs. 1, 6, 7 and 8). The dilatator operculi presents a relatively conservative morphology across the basal actinopterygians and teleosts in general. The muscle often exhibits a nearly bipennate architecture and an insertion partially or fully tendinous.

The morphology of the levator arcus palatini is also relatively constant across ray-finned fishes. The muscle usually presents a narrow and often tendinous origin primarily from the postorbital region of the autosphenotic and a broader insertion on the lateral face of the hyomandibula, metapterygoid, and occasionally some adjacent suspensorial bones (Figs. 1, 6, 7 and 8). A levator arcus palatini with those features may be partially subdivided. In polypteriforms this subdivision occurs in a nearly parasagittal plane, forming a pars externa and a pars interna (Fig. 1). These sections could not be reliably homologized with the partes anterior and posterior that characterize the levator arcus palatini of the examined holosts and hiodontiforms (Figs. 6, 7 and 8; see Character 12). Nomenclatures for these muscle sections are surrounded by some confusion, inasmuch as the name levator arcus palatini has been ambiguously applied to either one, another, or both subdivisions (see synonymies in “Results”). We followed the latter option because it minimizes the conflicts and unifies the muscle terminology of basal actinopterygians with that of teleosts [1].

Elopiforms presents a levator arcus palatini with a main muscle section compatible with the foregoing generalized characterization, i.e., a narrow origin on the autosphenotic and a broad insertion on the hyomandibula and metapterygoid (Figs. 8 and 9). However, the elopiform muscle exhibits up to two additional components associated with other skeletal elements. These additional muscle sections thus seem to represent expansions, rather than subdivisions of the primitive levator arcus palatini (as is the case of the partes interna/externa and anterior/posterior of the muscle of non-teleosts). Accordingly, in elopiforms the muscle portion corresponding to the plesiomorphic condition is herein defined as the pars primordialis of the levator arcus palatini. The additional sections are termed partes temporalis and pharyngealis and have no direct correspondence with any muscle subdivision found in other basal actinopterygians. Divisions similar to the elopiform partes temporalis and pharyngealis are paralleled in some highly-derived acanthomorphs (M. Pastana, pers. comm.). However, the pars pharyngealis of these derived taxa emerges from a narrow gap between the hyomandibula and metapterygoid, rather than between the hyomandibula and the preopercle as in *Elops*.

In the examined Polypteriformes and Acipenseriformes, the dorsalmost fibers of the constrictor mandibularis dorsalis form a third muscle, the spiracularis (Figs. 1, 3 and 5). This muscle exhibits varying degrees of differentiation. In some acipenseriforms, the spiracularis is represented by only a few fibers poorly differentiated from the remainder of the constrictor mandibularis dorsalis (Fig. 5). As a consequence, the muscle was apparently overlooked by some studies [22, 23, 25]. Nevertheless, identification of the spiracularis is unequivocal as it invariably associates with the connective tissue forming the anterior border of the spiracle. In polypteriforms the muscle is better differentiated from the dilatator operculi and has an additional attachment to the spiracular ossicles (Fig. 1a). The spiracularis is the last muscle to differentiate from the primordium of the constrictor mandibularis dorsalis during the development of *Polypterus senegalus* [19].

The constrictor hyoideus dorsalis forms the last set of facial muscles of ray-finned fishes (Figs. 1b, 2, 3, 4, 7, 8 and 9). The muscle is mostly laminar and usually connects the lateral region of the otic-temporal portion of the neurocranium with the medial faces of the hyomandibula and opercle. Up to three main muscles may differentiate from the constrictor hyoideus dorsalis in basal actinopterygians: the adductor hyomandibulae, adductor operculi, and levator operculi (Fig. 9). The adductor hyomandibulae is basically defined by its anterior placement and insertion on the hyomandibula and, occasionally, adjacent ossifications of the palatoquadrate (see following paragraphs). The levator operculi is the posteriormost component of the constrictor hyoideus dorsalis and inserts along the dorsoposterior region of the medial face of the opercle. The adductor operculi lies between the two former muscles and inserts primarily on the anterodorsal region of the medial face of the opercle, proximate to its articulation with the hyomandibula. The degree of separation between these three muscles is highly variable even among closely related taxa (e.g. *Elops* and *Megalops*) and possibly subject to intraspecific variation, inasmuch as distinct studies report different degrees of subdivisions in a same species (compare, for instance, the present description of the constrictor hyoideus dorsalis of *Amia calva* with those of Allis [27] and Lauder [18]).

Some nomenclatural confusion surrounds the use of the terms adductor hyomandibulae and adductor arcus palatini. In the plesiomorphic condition for ray-finned fishes, the portion of the constrictor hyoideus dorsalis associated with the hyopalatine arch attaches solely to the hyomandibula (Figs. 2 and 9). As a consequence of this attachment, most previous studies on basal actinopterygians refer to this muscle as the adductor hyomandibularis [19, 20, 27] or, in its most modern orthography, adductor hyomandibulae [1, 18, 26]. In teleosts, however, the same muscle is more commonly referred to as the adductor arcus palatini [1, 47]. Basal teleosts retain the primitive attachment restricted to the hyomandibula (e.g. elopiforms; Fig. 9), but most teleosts have the muscle anteriorly expanded with additional insertions on the metapterygoid and, often, other elements of the palatoquadrate [1, 4, 47]. Nevertheless, the set of fibers attached to the hyomandibula may be separated from those inserting on the elements of the palatoquadrate and, in those instances, the two divisions are then termed adductor hyomandibulae and adductor arcus palatini, respectively [1, 4]. To further complicate the issue, some taxa have the anteriormost portion of the muscle further differentiated and then named extensor tentaculi (most Siluriformes [4]) or retractor arcus palatini (some Acanthuriformes and Tetraodontiformes [1–3]). The resulting nomenclatural confusion is obvious, with the name adductor arcus palatini having been ambiguously applied to: (1) the short, plesiomorphic muscle attached solely to the hyomandibula and corresponding to the adductor hyomandibulae of basal actinopterygians (e.g., Elopiformes [1]); (2) the whole expanded muscle attached to both hyomandibula and palatoquadrate and thus encompassing the primitive adductor hyomandibulae (most teleosts [1, 47]); (3) the anteriormost portion of the muscle attached solely to the palatoquadrate and separated from the posterior adductor hyomandibulae (e.g. some Clupeiformes, Cypriniformes, Osteoglossiformes, Perciformes, Siluriformes, and Tetraodontiformes [1, 2, 4, 48, 49]); or (4) an intermediate portion of the muscle located between the anteriormost retractor arcus palatini or extensor tentaculi and the posteriormost adductor hyomandibulae (e.g., some Acanthuriformes, Siluriformes, and Tetraodontiformes [1, 2, 4, 49, 50]).

To eliminate these nomenclatural incoherencies and minimize the unnecessary proliferation of muscle names, we suggest to dismiss the terms adductor arcus palatini, extensor tentaculi, retractor arcus palatini, and other derivatives (cf. [4, 51]). All these terms are needless because they are, often ambiguously, used to refer to particular modifications of the primitive adductor hyomandibulae. Attribution of alternative muscle names according to specific bone associations proved to result in chaotic anatomical terminologies, such as the alphanumeric nomenclature applied to the teleostean adductor mandibulae [6, 28]. We accordingly propose the universal use of the term adductor hyomandibulae, regardless of its additional association with elements of the palatoquadrate. When necessary, subdivisions of the adductor hyomandibulae may be simply designated as a partes or sections of the adductor hyomandibulae. As in the case of the elopiform levator arcus palatini discussed above, the muscle section corresponding to the primitive condition (i.e., insertion on the hyomandibula only) may be designated pars primordialis of the adductor hyomandibulae. The additional separate sections could be termed, for example, pars pterygoidea (inserting on the metapterygoid and endopterygoid) and pars palatina (inserting on the palatine; = extensor tentaculi). Simple combinations to designate compound sections are also feasible, such as pars pterygo-palatina and pars pterygo-primordialis. Other possibilities of naming the partes of the adductor hyomandibulae may be hypothesized and the most appropriate terminology could vary in taxa with a highly modified musculature. The universal use of the term adductor hyomandibulae also simplifies the designation of the compound muscle often formed by the fusion of the adductor hyomandibulae and the adductor operculi, which can be termed adductor hyo-operculi (Fig. 2).

In conclusion, the nomenclatural adaptations herein proposed eliminate ambiguity and preserve the information about the homologies of the involved muscle components. Such an approach seems to be the most appropriate for comparative studies of muscles with complex evolutionary histories, such as the adductor hyomandibulae and adductor mandibulae [6, 28].

### Phylogenetic inferences

**Fig. 10:**
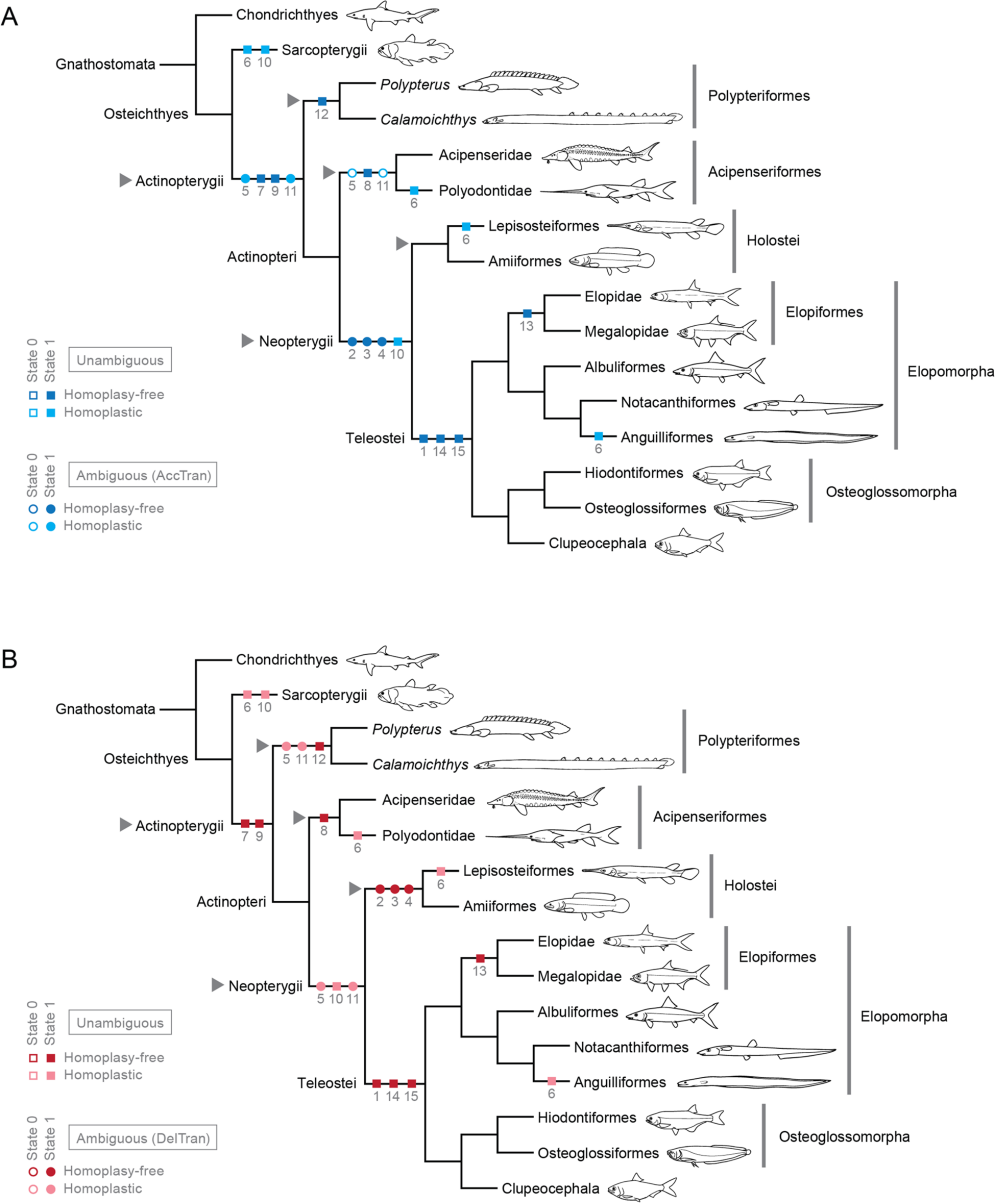
Maximum parsimony optimizations of the identified character states of the facial musculature superimposed on cladogram of basal gnathostomes [10, 52, 53]. Characters numbered as in text. Arrowheads indicate clades with synapomorphies affected by alternative optimizations. **a** Accelerated transformation (AccTran). **b** Delayed transformation (DelTran)

The following characters from the facial musculature are identified as phylogenetically informative for higher-level relationships of basal actinopterygians. These characters are optimized on a phylogenetic tree that synthesizes the currently accepted hypotheses of relationships among basal ray-finned fishes (Fig. 10) [10, 52, 53]. Conditions unique to a single examined species are not included in this analysis.

#### Character 1. Adductor mandibulae, segmentum buccalis; occurrence: (0) present, (1) absent

The presence of a segmentum buccalis is apparently primitive for bony fishes, as it is found in generalized cartilaginous fishes [32, 34], coelacanths [38, 39, 54], lungfishes [14, 17, 40–46], polypteriforms (Fig. 1), acipenseriforms (Figs. 3 and 4), and holosts (Figs. 6 and 7). Teleosts apomorphically lost the segmentum buccalis (Fig. 10), so that the segmentum facialis is the only component of the adductor mandibulae associated with the palatoquadrate and neurocranium in these fishes (Fig. 8).

Lauder [55] detected the absence in teleosts of a portion of the adductor mandibulae plesiomorphically found in gnathostomes. He postulated the plesiomorphic presence of four major subdivisions in the adductor mandibulae of the Osteichthyes: suborbital, medial, posterolateral, and intramandibular. Among these, the suborbital division would have been lost at the base of the Teleostei. However, the author failed to correctly identify the homology of the missing muscle division. Rather than the segmentum buccalis, Lauder’s [55] suborbital division corresponds to the pars orbitalis of *Amia* and *Lepisosteus* and to the segmentum facialis of *Polyodon*. He also erroneously homologized the segmentum buccalis of *Polypterus* with the pars sphenoidalis of holosts and the pars stegalis of teleosts (= his medial division).

#### Character 2. Adductor mandibulae, segmentum buccalis, pars sphenoidalis; degree of subdivision: (0) undivided, (1) subdivided into pars superficialis and pars profunda

The pars sphenoidalis of polypteriforms is completely undivided (Fig. 1) and, in acipenseriforms, the segmentum buccalis is undifferentiated into partes sphenoidalis and orbitalis (Figs. 3 and 4). Amiiforms and lepisosteiforms have the pars sphenoidalis clearly subdivided into superficialis and profunda subsections (Figs. 6 and 7). Teleosts lack a segmentum buccalis (Fig. 8; Character 1) and, therefore, the present character is inapplicable for the group. As a result, the sphenoidalis subdivided into partes superficialis and profunda may be equally optimized as a synapomorphy for either the Neopterygii (AccTran; Fig. 10a) or Holostei (DelTran; Fig. 10b).

#### Character 3. Adductor mandibulae, segmentum buccalis, pars orbitalis; degree of subdivision: (0) undivided, (1) subdivided into pars externa and pars interna

As in the case of the pars sphenoidalis discussed above, the pars orbitalis is undivided in polypteriforms and acipenseriforms (Figs. 1, 3 and 4) and secondarily subdivided into partes externa and interna in holosts (Figs. 6 and 7). Uniquely in *Amia*, the pars externa loses its connection with the lower jaw, being confined to the palatoquadrate. The present character is inapplicable for teleosts because they lack a segmentum buccalis (Character 1). Consequently, State 1 is ambiguously optimized as synapomorphic for either holosts (DelTran; Fig. 10b) or neopterygians (AccTran; Fig. 10a).

Lauder [18, 55] correctly homologized the subdivided segmentum buccalis of *Amia* and *Lepisosteus* (= his anterior or suborbital division of the adductor mandibulae). However, the author inexplicably omitted *Lepisosteus* from his phylogeny in Lauder [55] and erroneously considered the subdivided buccalis as an autapomorphy for *Amia*.

#### Character 4. Adductor mandibulae, segmentum buccalis, pars orbitalis; relative position: (0) posteroventral to eyeball, (1) anteroventral to eyeball

Generalized Chondrichthyes [32, 34], Coelacanthiformes [38, 39, 54], Dipnoi [14, 17, 40–46], Polypteriformes (Fig. 1), and Acipenseriformes (Figs. 3 and 4) have the entire segmentum buccalis confined to a region posteroventral to the eyeball. In holosts this muscle segment is much expanded anteriorly, so that its pars orbitalis is located anteroventral to the eyeball in adults (Figs. 6 and 7). This rostral expansion is also observed during the ontogenesis of the segmentum buccalis of both amiiforms and lepisosteiforms [16, 17, 26]. As in the two previous characters, optimization of this feature is ambiguous because of the absence of the segmentum buccalis in teleosts (Character 1). The pars orbitalis of the segmentum buccalis located anteroventral to the eyeball may be considered a synapomorphy for either Holostei (DelTran; Fig. 10b) or Neopterygii (AccTran; Fig. 10a).

#### Character 5. Adductor mandibulae, segmentum facialis; attachment to the hyoid arch: (0) absent, (1) present

Primitively in gnathostomes, the origin of the segmentum facialis of the adductor mandibulae is restricted to the palatoquadrate and, often, neurocranium. This configuration is found in most cartilaginous fishes [32, 34–37], coelacanths [38, 39, 54], lungfishes [14, 17, 40–46], and acipenseriforms (Figs. 3 and 4). Polypteriforms and neopterygians have the segmentum facialis of the adductor mandibulae posteriorly expanded and reaching also the hyomandibula and, eventually, other adjacent ossifications not derived from the palatoquadrate (e.g., preopercle and symplectic; Figs. 1, 6, 7 and 8). Under AccTran optimization, such a condition is optimized as a synapomorphy for Actinopterygii, with a reversal in Acipenseriformes (Fig. 10a); under DelTran, it appears independently in Polypteriformes and Neopterygii (Fig. 10b).

#### Character 6. Adductor mandibulae, segmentum mandibularis; occurrence: (0) present, (1) absent

The segmentum mandibularis is present in cartilaginous fishes and most basal actinopterygians, but is lacking in *Polyodon* and *Lepisosteus*. Such absences are optimized as independent losses in each taxon (Fig. 10). Additional losses of the same muscle segment occurred multiple times during the radiation of the Teleostei [1, 6, 28] and at the base of the Sarcopterygii [14, 17, 38–46, 54] (Fig. 10).

Lauder [55] surprisingly reported the presence of a segmentum mandibularis (his intramandibular division) in *Polyodon*. This finding is contradicted by the present survey, as well as all other previous myological studies of this taxon [14, 15, 25].

#### Character 7. Constrictor mandibularis dorsalis; attachment to the hyoid arch: (0) absent, (1) present

Generalized cartilaginous fishes [32, 34–37] and coelacanths [38, 39, 54] have a constrictor mandibularis dorsalis originating from the neurocranium and inserting solely on the palatoquadrate. In all ray-finned fishes, this muscle expands posteriorly so as to acquire an additional insertion on the hyomandibula and, occasionally, other elements associated with the hyoid arch (Figs. 1, 3, 4, 6, 7, 8 and 9). This condition is therefore hypothesized to be a synapomorphy for the Actinopterygii (Fig. 10).

Lungfishes completely lack a constrictor mandibularis dorsalis [14, 17, 40–43] and are thus inapplicable for the present character.

#### Character 8. Constrictor mandibularis dorsalis; attachment to the palatoquadrate: (0) present, (1) absent

As described in Character 7, in the gnathostome plesiomorphic condition the constrictor mandibularis dorsalis inserts onto the palatoquadrate and, eventually, other elements of the viscerocranium (Figs. 1, 6, 7, 8 and 9) [32, 34–39, 54]. The plesiomorphic connection with the palatoquadrate is secondarily lost in the Acipenseriformes (Fig. 10) and the entire muscle inserts solely on the hyoid arch (Figs. 3 and 4).

This character is inapplicable for dipnoans as they lack a constrictor mandibularis dorsalis [14, 17, 40–43].

#### Character 9. Constrictor mandibularis dorsalis; insertion on the lateral face of the palatoquadrate: (0) absent, (1) present

In the plesiomorphic gnathostome condition, the constrictor mandibularis dorsalis inserts along the dorsal border and medial face of the palatoquadrate. This morphology is observed in generalized chondrichthyans [32, 34–37] and in coelacanths [38, 39, 54]. Actinopterygians, in contrast, have this muscle inserting entirely or mostly on the lateral face of the elements of the palatoquadrate (Figs. 1, 6, 7, 8 and 9). Such a condition is interpreted to be an additional synapomorphy for the Actinopterygii (Fig. 10).

The present character is inapplicable for lungfishes, which lack a constrictor mandibularis dorsalis [14, 17, 40–43], and for acipenseriforms that have the muscle apomorphically inserting only on the hyoid arch (Character 7).

#### Character 10. Constrictor mandibularis dorsalis, distinguishable spiracularis: (0) present, (1) absent

The spiracularis is represented by a dorsalmost set of fibers of the constrictor mandibularis dorsalis that subtly associates with the connective tissues forming the anterior border of the spiracular opening of acipenseriforms and polypteriforms (Figs. 1, 3 and 5). The muscle is poorly differentiated, but unequivocally identified in the former group. Polypteriforms exhibit a conspicuous spiracularis well separated from the remainder of the constrictor mandibularis dorsalis and with an additional insertion on the spiracular ossicles. The presence of a spiracularis is likely primitive for bony fishes because it is found in most generalized cartilaginous fishes. In chondrichthyans that muscle is represented by a posterodorsal set of fibers around the anterior border or the spiracular opening, with varying degrees of differentiation from the main bulk of the constrictor mandibularis dorsalis [32, 34–37]. A distinguishable spiracularis and the associated external spiracular opening are absent in all neopterygians, as well as in living coelacanths and lungfishes [14, 17, 38–43, 46, 54, 56]. With the available data, it is impossible to confidently assert whether the muscle fibers corresponding to the spiracularis are indeed absent or merely undifferentiated from the remainder of the constrictor mandibularis dorsalis in the Neopterygii and Sarcopterygii. In any event, the loss or non-differentiation of the spiracularis is most parsimoniously interpreted as evolving independently in neopterygians and sarcopterygians (Fig. 10).

Diogo [57, 58] mentioned that “no spiracularis was found in the *Polypterus* specimens or in any other osteichthyan specimens examined in the present work”. This finding is refuted by the present analysis (Figs. 1, 3 and 5) and several previous studies [13, 14, 19, 20]. The spiracularis was unequivocally identified in all six specimens of polypteriforms and the three specimens of acipenseriforms herein examined.

#### Character 11. Constrictor mandibularis dorsalis; differentiation into levator arcus palatini and dilatator operculi: (0) absent, (1) present

In all actinopterygians except acipenseriforms, the main bulk of the constrictor mandibularis dorsalis is differentiated into at least two major muscles. The anterior one is the levator arcus palatini, which retains the plesiomorphic actinopterygian insertion on the palatoquadrate and hyoid arch (Figs. 1, 6, 7 and 8; Character 7). The second muscle is the dilatator operculi that acquires a novel insertion on the anterodorsal region of the opercle (Figs. 1, 6, 7 and 8). The differentiation of the dilatator operculi and its attachment to the opercle are not found elsewhere among extant jawed fishes, even in those taxa also exhibiting a dermal opercle (e.g., coelacanths and lungfishes [14, 17, 38–43, 54]). As mentioned, the constrictor mandibularis dorsalis of generalized cartilaginous fishes is a simple muscle with its origin on the neurocranium and insertion on the palatoquadrate and spiracular canal (Character 7) [32, 34–37]. Therefore, the differentiation of the constrictor mandibularis dorsalis into levator arcus palatini and dilatator operculi (and the consequent attachment to the opercle) is most parsimoniously optimized as a synapomorphy for the Actinopterygii, with a reversal in the Acipenseriformes (AccTran; Fig. 10a). This reversal may be coupled with the hypothesized loss of the opercle in extant acipenseriforms [59–67]. State 1 could alternatively be optimized as independently evolving in the Polypteriformes and Neopterygii (DelTran; Fig. 10b), but this scheme implies in the unlikely parallelism of both the differentiation of the two muscles and the attachment of the dilatator operculi on the opercle.

#### Character 12. Constrictor mandibularis dorsalis, levator arcus palatini; differentiation into partes interna and externa: (0) absent, (1) present

Polypteriforms have a levator arcus palatini subdivided along a parasagittal plane forming the partes interna and externa (Fig. 1). This muscle is also subdivided in holosts and hiodontiforms, but the subdivision occurs in a nearly transverse plane resulting in the formation of a pars anterior and a pars posterior (Figs. 6 and 7). As a consequence, the two types of subdivision of the levator arcus palatini cannot be reliably hypothesized to be homologous. Moreover, the partes interna and externa of polypteriforms exhibit unique features, such as the arched tendon for the insertion of the former subdivision (Fig. 1). Therefore, the differentiation of the levator arcus palatini into partes externa and interna with those particular features is optimized as a synapomorphy for the Polypteriformes (Fig. 10).

Elopiforms exhibit a distinct condition of the levator arcus palatini in which additional muscle sections result from expansions, rather than the subdivision, of the primitive muscle (see Character 13). The aforementioned differentiation of the levator arcus palatini into partes anterior and posterior (Figs. 6 and 7) is not restricted to holosts and hiodontiforms, being also found in other teleosts. Because the precise distribution of this feature could not be reliably determined, it was not included in the present analysis. The levator arcus palatini as defined in Character 11 is absent in non-actinopterygians, which are therefore treated as inapplicable for the present character.

#### Character 13. Constrictor mandibularis dorsalis, levator arcus palatini, pars temporalis; occurrence: (0) absent, (1) present

Elopiforms have a levator arcus palatini with a posterior expansion that laterally covers most of the dilatator operculi (Figs. 8 and 9). This muscle section attaches to the borders of the dilatator fossa and is herein termed pars temporalis. Presence of this section is unique among basal ray-finned fishes. A muscle division similar to the pars temporalis occurs only in a few specialized carangiforms and scombriforms (M. Pastana, pers. comm.; AD, pers. obs.). Because these taxa are deeply nested within the Percomorphacea, a highly derived teleostean clade [12, 52], the presence of a pars temporalis can be confidently optimized as an unequivocal synapomorphy for the Elopiformes (Fig. 10).

Although the order Elopiformes has long been recognized in taxonomic classifications, the group is poorly defined in phylogenetic terms and occasionally not resolved as monophyletic in morphological analyses [68, 69]. As discussed by Wiley & Johnson [12] and Johnson & Britz [70], all previous morphological synapomorphies proposed for the order are ambiguous, highly homoplastic, or dependent of outgroup choice. The possession of a pars temporalis of the levator arcus palatini is, therefore, the first unequivocal morphological synapomorphy known to date for the Elopiformes.

Non-actinopterygians lack a differentiated levator arcus palatini (Character 11) and are, therefore, inapplicable for the present character.

#### Character 14. Opercle, dilatator process; occurrence: (0) absent, (1) present

Teleosts present a small reinforced expansion on the opercle that collects part of the muscular and tendinous fibers of the dilatator operculi (Fig. 8: arrow). This expansion is invariably projected dorsolaterally and is herein defined as the dilatator process of the opercle. The degree of development of this process greatly varies, but its presence is almost universal in teleosts. In at least some taxa, the degree of expansion of the process seems to vary according to the overall size of the dilatator operculi [4]. Basal actinopterygians lack a dorsolaterally projected process in the opercle serving as insertion site for the dilatator operculi. The possession of a dilatator process is thus synapomorphic for teleosts (Fig. 10).

The present character is inapplicable for extant sarcopterygians, chondrichthyans and acipenseriforms because they lack a dilatator operculi (Character 11) and the latter two groups additionally lack a dermal opercle [59–67].

#### Character 15. Opercle, adductor crest; occurrence: (0) absent, (1) present

The adductor operculi invariably inserts on the anterodorsal region of the medial face of the opercle (Figs. 2 and 9). Teleosts have a horizontal crest on the opercle that serves as a reinforced site for the insertion of that muscle (Fig. 9: arrow). This structure, herein named adductor crest, emerges from the elliptical depression that articulates with the hyomandibular condyle and gradually dissipates posteriorly. As expected, taxa with hypertrophied adductores operculorum exhibit proportionally expanded adductor crests [4]. In many teleosts, the adductor crest and associated articular depression are typically the first structures to ossify during opercle ontogenesis [71–73]. Although non-teleost actinopterygians do have an adductor operculi, they lack an associated adductor crest in the opercle. A comparable crest is also absent in sarcopterygian fishes [39, 41–46]. The presence of an adductor crest is consequently optimized as a synapomorphy for the Teleostei (Fig. 10).

Extant chondrichthyans and acipenseriforms lack a dermal opercle and are accordingly inapplicable for this character [59–67].

### Remarks on the upper jaw of acipenseriforms

As in other actinopterygians, the largest bone at the lateral border of the upper jaw of acipenseriforms has been traditionally identified as the maxilla [14, 25, 31, 62, 74–76]. However, Findeis [77] proposed in a short proceeding abstract that this ossification would actually represent a dermopalatine, and the maxilla would have been lost in acipenseriforms. The author justifies his conclusion arguing that the typical actinopterygian maxilla lacks “extensive contact” with the palatoquadrate cartilage and the acipenseriform bone in question “arises from two independent membrane ossifications that invest the palatoquadrate cartilage”. Findeis’ [77] hypothesis has been followed by most subsequent studies and became the predominant view in the recent acipenseriform literature [23, 63–67, 78]. However, the relationships between the adductor mandibulae and the surrounding skeleton strongly disagrees with Findeis’ [77] proposition, as discussed below.

**Fig. 11:**
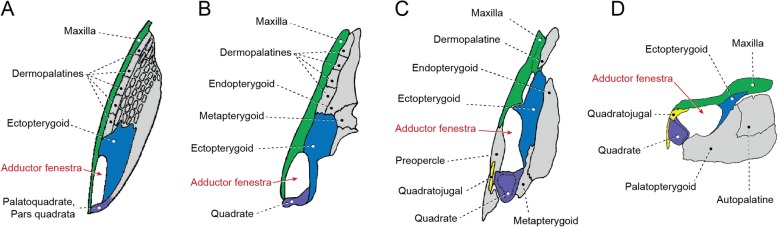
Schematic reconstruction of palatal arch and associated bones of basal actinopterygians; right side, ventral view. **a** †*Cheirolepis trailli* (modified from [79]); **b** †*Mimia toombsi* (modified from [80]); (**c**) *Polypterus bichir* (modified from [80]); (**d**) *Acipenser brevirostrum* (modified from [67])

In bony fishes the facial and buccal segments the adductor mandibulae pass through a gap, herein termed the adductor fenestra, in order to insert onto the lower jaw. Actinopterygians have this fenestra delimited posteriorly by the quadrate (or pars quadrata of the palatoquadrate), anteromedially by the ectopterygoid, and anterolaterally by the maxilla (Fig. 11). Other bones may marginally border the adductor fenestra (e.g. quadratojugal), but the dermopalatine is never associated with this gap. Such a configuration is found in all basal actinopterygians, including both extinct and living forms [79–81]. The palatoquadrate of acipenseriforms undergoes a medial rotation in comparison with that of other actinopterygians, but a similar adductor fenestra is easily identifiable (Fig. 11d; see also fig. 134 of Hilton et al. [67]). This fenestra is also bordered by the quadrate, ectopterygoid, and an anterolateral bone that, accordingly, should be most obviously homologized with the maxilla of other actinopterygians. Moreover, all these basal actinopterygians have the anterior portion of the maxilla with an “extensive contact” with the palatal arch (Fig. 11) [79–81]. Therefore, contrary to Findeis’ [77] argumentation, that feature actually supports, rather than refutes, the homologization of the acipenseriform ossification in question with the maxilla. A movable maxilla with limited contact with the palatoquadrate is a derived condition typical of neopterygians (e.g., *Amia* and teleosts; Figs. 7 and 8).

The ontogenetic argument provided by Findeis’ [77] also does not support his conclusion. The acipenseriform bone in question begins to ossify along the anterolateral wall of the main bulk of the adductor mandibulae, far removed from the cartilaginous palatoquadrate (see fig. 30 of Sewertzoff [31] and fig. 4d of Warth et al. [78]). The bone subsequently expands anteromedially and then acquires a closer connection with the anterior portion of the palatoquadrate. The ontogenesis of the dermopalatine in other basal actinopterygians is remarkably different: the bone begins to ossify anteriorly to the adductor fenestra and alongside the lateral wall of the palatoquadrate [82]. Furthermore, in no known actinopterygian is the dermopalatine partially separated from the remainder of the palatal arch by a large fenestra traversed by a muscle (adductor mandibulae).

In conclusion, the hypothesis that the main external bone at the upper jaw of acipenseriforms corresponds to a dermopalatine requires the following evolutionary changes: (1) loss of the maxilla; (2) dermopalatine beginning its ossification far removed from the palatoquadrate; and (3) dermopalatine posterolaterally expanded, so that it occupies the same basic position as the maxilla and forms the anterolateral border of the adductor fenestra. Homologization of the referred bone with the maxilla, in turn, requires only a single step: (1) loss of the dermopalatine. The latter hypothesis is more parsimonious and, therefore, preferable over the former.

## Conclusions

Twenty main muscle divisions are identified in the facial musculature of basal ray-finned fishes. All these components derive evolutionarily and ontogenetically from three primordial muscles, the adductor mandibulae, constrictor mandibularis dorsalis, and constrictor hyoideus dorsalis. Clarification of the muscle homologies resulted in the proposition of a unifying terminology for the facial musculature of all actinopterygians. Sixteen new characters associated with the facial musculature are identified as informative for the higher-level phylogeny of bony fishes. New, unequivocal synapomorphies are proposed for Actinopterygii, Neopterygii, Teleostei, Polypteriformes, Acipenseriformes, and Elopiformes. Elopiformes has long been recognized in taxonomic classifications and in molecular phylogenetic studies, but until now lacked unequivocal morphological synapomorphies. Myological support for the Holostei is ambiguous and recovered only under DelTran optimization. These findings demonstrate the underexplored potential of the myology as a source of phylogenetic information and provide a background for future studies on the facial musculature of both derived actinopterygians and sarcopterygians.

## Methods

### Specimens

Examined material is listed in Table 1 and is deposited in the following public institutions: Laboratório de Ictiologia de Ribeirão Preto, Universidade de São Paulo, Brazil (LIRP); Museu de Zoologia da Universidade de São Paulo, Brazil (MZUSP); National Museum of Natural History, Smithsonian Institution, USA (USNM); and Virginia Institute of Marine Science, USA (VIMS). Access to material of these collections and permission for dissections were duly authorized by the respective curators. Additional examined comparative material of the Teleostei is listed in Datovo & Vari [6, 28] and Datovo et al. [83].

### Techniques

Preserved specimens were double-stained for cartilage and bone prior to dissections following the methodology of Datovo & Bockmann [4]. Dissections were made under stereomicroscope. Anatomical drawings were based on photographs and direct stereomicroscopic observations of specimens in order to capture fine anatomical details. Drawings are bidimensional and were produced with a Wacom Intuos4 pen tablet (Wacom Company, Ltd., Tokyo, Japan). Outlines were generated in Adobe Illustrator CC and the shading and coloring in Adobe Photoshop CC (Adobe Systems, San Jose, CA, USA).

### Anatomical terminology

In the anatomical descriptions, the term insertion refers to the attachment of the muscle to the structure (usually a bone) that presumably moves (or moves more intensely) during its contraction; origin is defined as the opposite muscle attachment to the stationary (or less movable) skeletal element [1]. Musculous attachment (origin or insertion) is when the muscles fibers attach directly to the skeleton without the mediation of any macroscopically evident tendon. In the tendinous attachment, the muscle fibers converge to a macroscopically evident tendon, which in turn attaches to the skeleton. In some instances, the attachment of a muscle is partially musculous and partially tendinous.

Osteological terminology primarily follows Grande & Bemis [84], Hilton [85], Grande [53], Hilton et al. [67], and Claeson et al. [86], but the following exceptions should be highlighted. The term sphenotic is retained only for the compound ossification formed by the fusion of the autosphenotic (chondral origin) and dermosphenotic (dermal origin; = posterodorsal most infraorbital), such as in *Polypterus* (Fig. 1). As a consequence, and in order to achieve correspondence between terminology and homology, the bone formed only by the chondral element but traditionally referred to as the “sphenotic” in most neopterygians is accordingly named autosphenotic (Figs. 6, 7 and 8); the element derived solely from the dermal ossification is obviously termed dermosphenotic (Fig. 3). The acipenseriform bone referred to as the “dermopalatine” by some studies is herein interpreted to correspond to the maxilla, as discussed in the section “Remarks on the upper jaw of acipenseriforms”.
